# Hydrogel-chitosan and polylactic acid-polycaprolactone bioengineered scaffolds for reconstruction of mandibular defects: a preclinical *in vivo* study with assessment of translationally relevant aspects

**DOI:** 10.3389/fbioe.2024.1353523

**Published:** 2024-07-15

**Authors:** Marco Ferrari, Stefano Taboni, Harley H. L. Chan, Jason Townson, Tommaso Gualtieri, Leonardo Franz, Alessandra Ruaro, Smitha Mathews, Michael J. Daly, Catriona M. Douglas, Donovan Eu, Axel Sahovaler, Nidal Muhanna, Manuela Ventura, Kamol Dey, Stefano Pandini, Chiara Pasini, Federica Re, Simona Bernardi, Katia Bosio, Davide Mattavelli, Francesco Doglietto, Shrinidh Joshi, Ralph W. Gilbert, Piero Nicolai, Sowmya Viswanathan, Luciana Sartore, Domenico Russo, Jonathan C. Irish

**Affiliations:** ^1^ Guided Therapeutics (GTx) Program International Scholarship, University Health Network (UHN), Toronto, ON, Canada; ^2^ Section of Otorhinolaryngology-Head and Neck Surgery, Department of Neurosciences, University of Padua, Padua, Italy; ^3^ Unit of Otorhinolaryngology-Head and Neck Surgery, Azienda Ospedale-Università di Padova, Padova, Italy; ^4^ Artificial Intelligence in Medicine and Innovation in Clinical Research and Methodology (PhD Program), Department of Clinical and Experimental Sciences, University of Brescia, Brescia, Italy; ^5^ Guided Therapeutics (GTx) Program, Techna Institute, University Health Network, Toronto, ON, Canada; ^6^ Department of Otorhinolaryngology, Head & Neck Surgery, Nuovo Santo Stefano Civil Hospital, Prato, Italy; ^7^ Osteoarthritis Program, Schroeder Arthritis Institute, Krembil Research Institute, Institute of Biomedical Engineering, University Health Network, University of Toronto, Toronto, ON, Canada; ^8^ Princess Margaret Cancer Centre, Toronto General Hospital, Department of Otolaryngology-Head and Neck Surgery/Surgical Oncology, University Health Network, Toronto, ON, Canada; ^9^ Department of Otolaryngology, Head and Neck Surgery, Queen Elizabeth University Hospital, Glasgow, United Kingdom; ^10^ Department of Otolaryngology-Head and Neck Surgery, National University Hospital, Singapore, Singapore; ^11^ Head & Neck Surgery Unit, University College London Hospitals, London, United Kingdom; ^12^ Department of Otolaryngology-Head and Neck Surgery, Tel Aviv Sourasky Medical Center, Tel Aviv University, Tel Aviv, Israel; ^13^ STTARR Innovation Centre, University Health Network, Toronto, ON, Canada; ^14^ Human Technopole Foundation, Milan, Italy; ^15^ Department of Mechanical and Industrial Engineering, University of Brescia Via Branze, Brescia, Italy; ^16^ Department of Applied Chemistry and Chemical Engineering, Faculty of Science, University of Chittagong, Chittagong, Bangladesh; ^17^ Unit of Blood Diseases and Bone Marrow Transplantation, Department of Clinical and Experimental Sciences, ASST Spedali Civili, University of Brescia, Brescia, Italy; ^18^ Centro di Ricerca Emato-Oncologica AIL (CREA), ASST Spedali Civili, Brescia, Italy; ^19^ Unit of Otorhinolaryngology-Head and Neck Surgery, ASST Spedali Civili of Brescia, Brescia, Italy; ^20^ Department of Medical and Surgical Specialties, Radiological Sciences, and Public Health, University of Brescia, Brescia, Italy; ^21^ Neurosurgery Unit, Fondazione Policlinico Universitario Agostino Gemelli, Rome, Italy; ^22^ Catholic University School of Medicine, Rome, Italy

**Keywords:** bone regeneration, porous scaffold, osteogenesis, mesenchymal stromal cells, tissue engineering, head and neck, reconstruction, mandible

## Abstract

**Background:** Reconstruction of mandibular bone defects is a surgical challenge, and microvascular reconstruction is the current gold standard. The field of tissue bioengineering has been providing an increasing number of alternative strategies for bone reconstruction.

**Methods:** In this preclinical study, the performance of two bioengineered scaffolds, a hydrogel made of polyethylene glycol-chitosan (HyCh) and a hybrid core-shell combination of poly (L-lactic acid)/poly ( ε -caprolactone) and HyCh (PLA-PCL-HyCh), seeded with different concentrations of human mesenchymal stromal cells (hMSCs), has been explored in non-critical size mandibular defects in a rabbit model. The bone regenerative properties of the bioengineered scaffolds were analyzed by *in vivo* radiological examinations and *ex vivo* radiological, histomorphological, and immunohistochemical analyses.

**Results:** The relative density increase (RDI) was significantly more pronounced in defects where a scaffold was placed, particularly if seeded with hMSCs. The immunohistochemical profile showed significantly higher expression of both VEGF-A and osteopontin in defects reconstructed with scaffolds. Native microarchitectural characteristics were not demonstrated in any experimental group.

**Conclusion:** Herein, we demonstrate that bone regeneration can be boosted by scaffold- and seeded scaffold-reconstruction, achieving, respectively, 50% and 70% restoration of presurgical bone density in 120 days, compared to 40% restoration seen in spontaneous regeneration. Although optimization of the regenerative performance is needed, these results will help to establish a baseline reference for future experiments.

## Introduction

Reconstruction of mandibular bone defects following ablation or trauma of the head and neck is a surgical challenge. The reconstruction must provide adequate mechanical support, maintenance of basic physiological functions (*i.e.*, breathing, swallowing, speaking), and an acceptable esthetic profile. Currently, the gold standard for many of these defects is bone-containing free tissue transfer (*e.g.*, scapular tip flap, fibular flap, iliac crest flap) (Wallace et al., 2010; Gibber et al., 2015; Blumberg et al., 2019). Microvascular procedures provide optimal results thanks to the high viability of bone tissue. This characteristic renders re-vascularized bone-containing free flaps far more appealing than bone grafting, especially when radiotherapy is planned. On the other hand, such reconstructions are technically demanding, require high expertise, and can be remarkably time-consuming. In addition, donor site morbidity, although potentially minimal in expert hands, can be considered as a further unavoidable drawback of these techniques (Mureau et al., 2005; Momoh et al., 2011; Klinkenberg et al., 2013; Orlik et al., 2014; Oh et al., 2019; Patel et al., 2020).

Over the last decades, an increasing number of advancements from the field of bioengineering have opened the possibility of regeneration of bone, cartilage, and mucosa, arousing interest in several surgical specialties, including head and neck surgery (Gualtieri et al., 2021). Furthermore, it is clear that game-changing bioengineering advances are applicable to many clinical settings including surgical oncology, organ transplantation, trauma surgery, cardiovascular interventions, orthopedics, dentistry, and many others.

Bone tissue bioengineering relies on a “triad” of factors.

1) An adequate scaffold serving as temporary framework for new tissue formation;
2) Stem cells able to proliferate and differentiate in different lineages (*i.e.*, bone, cartilage);
3) Efficient biochemical or physical triggers able to induce and maintain the process of new tissue formation (Tollemar et al., 2016; Ho-Shui-Ling et al., 2018).

Several systematic reviews have highlighted the available materials (Pilipchuk et al., 2015; Hosseinpour et al., 2017; Kim et al., 2017; Roffi et al., 2017), stem cells (Cecilia and Maria, 2014; Pilipchuk et al., 2015; Roffi et al., 2017), and trigger factors (Roffi et al., 2017), along with several variants in terms of production, refinement, implementation, and combination of these fundamentals (Saeed et al., 2015; Kim et al., 2017). The remarkable quantity of preclinical data obtained *in vitro* and *in vivo* has been recently followed by a few but significant applications to in-human use, which reinforce the belief that this technology can be translated into clinical practice (Saeed et al., 2015; Kim et al., 2017).

Ideally, a scaffold intended for bone reconstruction conveys several properties: 1) it must be biocompatible, and thus not elicit excessive adverse reactions nor cause organ toxicity; 2) it must be bioresorbable and/or biodegradable; 3) the timing of resorption/degradation should be synchronous with that of new tissue formation; 4) the scaffold must have sufficient mechanical properties to temporarily substitute the missing bone; 5) it must be osteoinductive and osteoconductive by means of optimal porosity; 6) it should induce neovascularization (Albrektsson and Johansson, 2001; Lee et al., 2013; Ren et al., 2015; Ren et al., 2016; Tollemar et al., 2016; Yuan et al., 2018; Zhang et al., 2018).

Currently, a material with all these characteristics has not been discovered, even if some materials have demonstrated excellent performance for some characteristics. For instance, hydrogels are remarkably suitable for neovascularization, but mechanically inadequate to sustain bone mechanical functions, whereas polylactic acid (PLA) and polycaprolactone (PCL) are stiffer and more osteoinductive, but less prone to be vascularized. Consequently, the combination of various materials with complementary features could enable to create an ideal scaffold for complex reconstruction (Qu et al., 2015; Patel et al., 2018; Upadhyay et al., 2020). With this aim, a novel synthetic strategy to produce a mechanically strong gelatin-based hydrogel using poly (ethylene glycol) diglycidyl ether as a cross-linker has been developed (Dey et al., 2019a; Dey et al., 2020). This hydrogel with chitosan (HyCh) has been proven to efficiently support cell growth, osteo-differentiation, and mineralization (Dey et al., 2019a; Dey et al., 2019b; Re et al., 2019; Re et al., 2021); furthermore, it is able to trigger the osteogenic differentiation of hMSCs without external stimuli (Bernardi et al., 2020).

In addition to hydrogels alone, a three-dimensional integrated core-shell structure has been developed by grafting the softer bioactive HyCh-shell onto a stiffer thermoplastic porous core of poly (L-lactic acid)/poly (ε-caprolactone). The hybrid scaffolds, herein acronymized as PLA-PCL-HyCh, resulted in an exceptional improvement of mechanical properties compared to the pure hydrogel, closely mimicking both the stiffness and the morphology of bones. Furthermore, hybrid PLA-PCL-HyCh scaffolds showed excellent capability in supporting cell growth, osteogenic differentiation, and mineralization of bone marrow hMSCs (BM-hMSCs) (Dey et al., 2019a; Dey et al., 2019b; Re et al., 2019; Bernardi et al., 2020; Dey et al., 2020; Re et al., 2021; Sartore et al., 2022).

A pilot translational study assessing bone regeneration sustained by HyCh and PLA-PCL-HyCh polymer scaffolds in an *in vivo* animal model is presented herein. The study aims to: 1) evaluate the *in vivo* bone regenerative potential of materials developed by our research group (*i.e.*, HyCH and PLA-PCL-HyCh) (Dey et al., 2019a; Re et al., 2019; Sartore et al., 2022): 2) assess the safety of xenoimplantation of scaffolds seeded with human mesenchymal stromal cells (hMSC) in New Zealand rabbits; 3) investigate the effect of translationally relevant variables on the process of bone regeneration; 4) analyze the characteristics of new bone at the microarchitectural and immunohistochemical levels; and 5) establish a baseline bone regeneration model for future studies.

## Materials and methods

### Study design and summary

**FIGURE 1:**
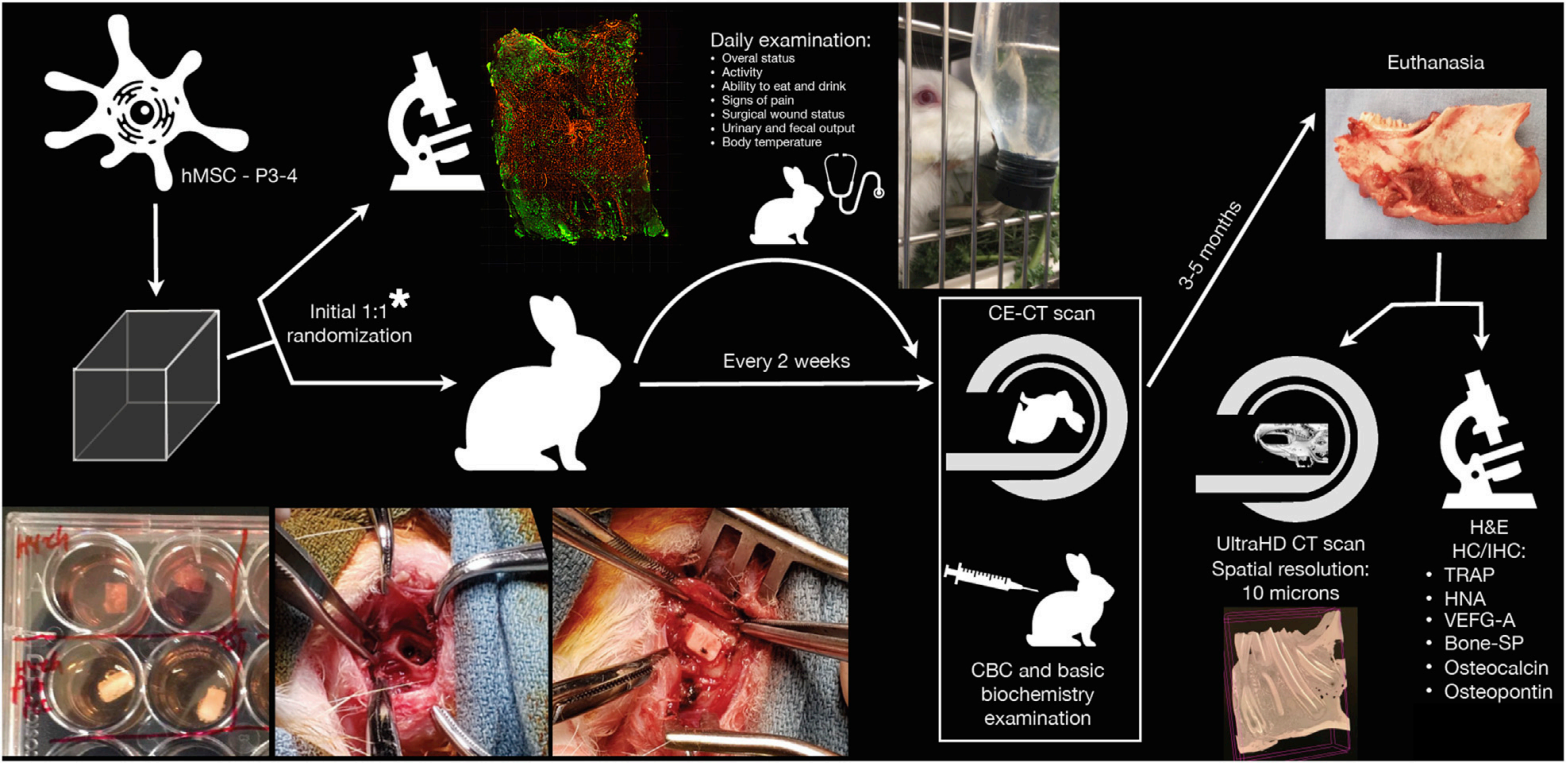
Schematic representation of the study workflow. *Randomization of bioengineered scaffolds was performed to ascertain scaffolds were effectively seeded with viable human mesenchymal stromal cells (hMSCs) at the time of surgery. CBC, complete blood count; CE-CT, contrast-enhanced computed tomography; H&E, hematoxylin-eosin; HC/IHC, histochemistry/immunohistochemistry; HNA, human nuclear antigen; P3-4, passage 3-to-4; SP, sialoprotein; TRAP, tartrate-resistant acid phosphatase; UltraHD, ultra-high-definition; VEGF-A, vascular-endothelial growth factor-A.

This preclinical study used immunocompetent male New Zealand rabbits (*Oryctolagus cuniculus*; body weight ≥3 kg) to analyze the regenerative properties of bioengineered scaffolds (HyCh and PLA-PCL-HyCh seeded with hMSCs) in non-critical-size mandibular defects. Two study phases were planned: 1) an *in vitro* phase, that aimed to verify the presence of viable hMSCs in the scaffold at the time of surgery; 2) a *in vivo* phase aimed to assess the safety of the experimental procedure and analyze the performance of bioengineered scaffold-based bone regeneration through multiple analyses (*i.e.*, *in vivo* and *ex vivo* radiological examinations and *ex vivo* histomorphological and immunohistochemical analyses). Spontaneous bone regeneration has been studied (*i.e.*, considering animals with identical size of mandibular defects with either no reconstruction or unseeded scaffold-reconstruction as “controls”). The following variables were analyzed: 1) type of the scaffold (HyCh *vs* PLA-PCL-HyCh); 2) dimension of defect (3–5 x 3 x 3 mm^3^ *vs* 15 × 3 × 3 mm^3^); 3) type of contamination of the surgical site (sterile transcervical inferior mandibulectomy *vs* contaminated transoral teeth-sparing mandibulectomy); 4) quantity of seeded hMSCs (1,000 cells/mm^3^ *vs* 2,000 cells/mm^3^ *vs* 3,000 cells/mm^3^). The study workflow is summarized in Figure 1.

### Polymer scaffold synthesis

Two different biocompatible and bioresorbable polymeric scaffolds were tested: HyCh and PLA-PCL-HyCh. HyCh is a highly porous and structurally stable hydrogel obtained by chemical crosslinking of poly (ethylene glycol) diglycidyl ether (PEG), gelatin (G), and chitosan (Ch). The material was prepared with a novel 2-step technique to increase the physical-mechanical stability of the scaffold: a first homogeneous phase reaction followed by freezing, freeze-drying, and a post-curing process. G, PEG and Ch content in the dry sample was 74.3%, 17.6%, and 8.1%, respectively (Dey et al., 2019a).

**FIGURE 2:**
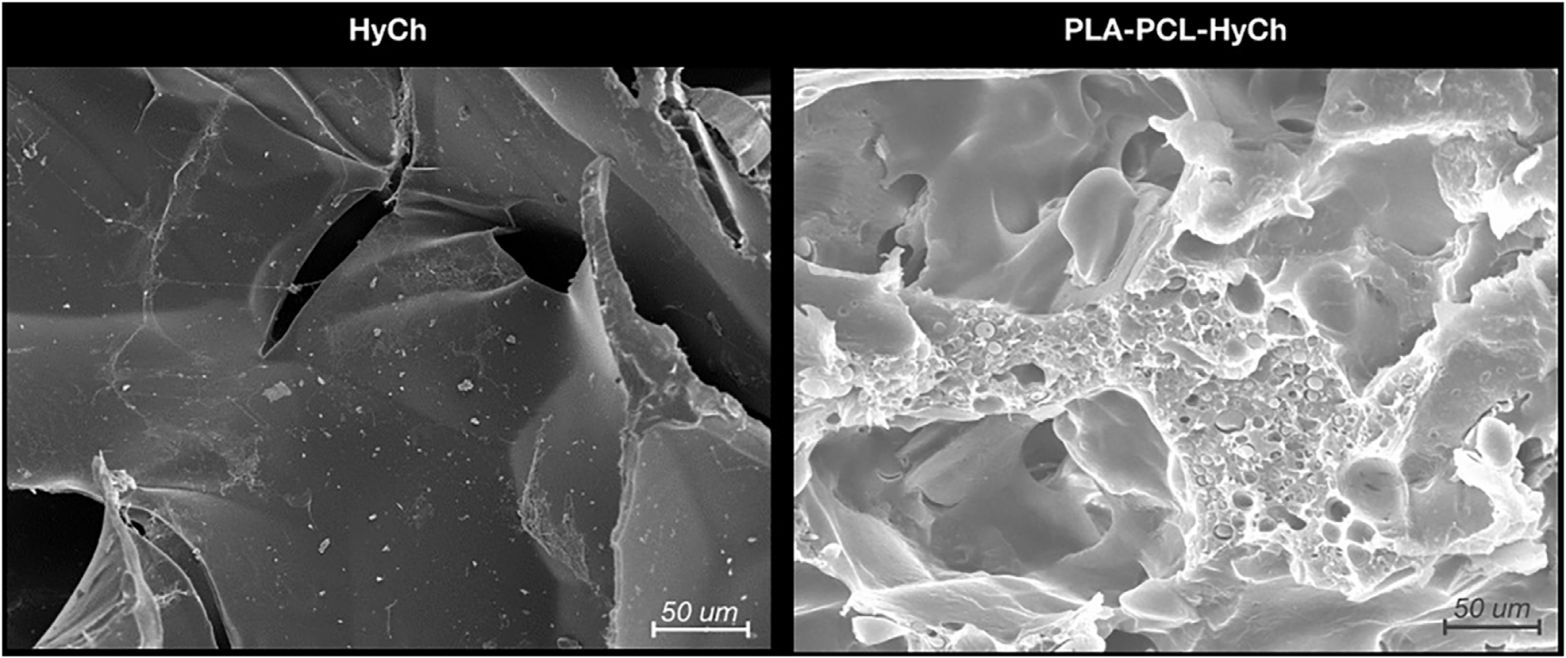
Microstructure of biomaterials (*i.e.*, hydrogel [HyCh] and the hybrid core-shell structure [PLA-PCL-HyCh]) as seen by scanning electron microscopy (SEM).

An innovative synthetic approach was adopted to develop a hybrid core-shell scaffold with a PLA-PCL rigid core and HyCh soft shell. An interconnected porous core was safely obtained, avoiding solvents or other chemical issues, by blending PLA, PCL, and leachable superabsorbent polymer particles. After particle leaching in water, the resulting porous core was grafted with HyCh to create a bioactive shell within its pores. The final amount of grafted HyCh was 3% by weight (Re et al., 2019; Sartore et al., 2022). Figure 2 shows the morphological analysis of cryogenically obtained cross sections for HyCh and PLA-PCL-HyCh scaffolds. Both materials revealed a highly interconnected irregular open pore morphology which is conducive to the infiltration of cells.

Both dry scaffolds were packed in polypropylene bags and sterilized by gamma irradiation with cobalt 60 gamma rays (dose: 27–33 kGy, according to UNI EN ISO 11,137- Sterilization of Healthcare Products) (ISO, 2022). The scaffolds were developed and produced at the Department of Mechanical and Industrial Engineering, University of Brescia (Brescia, Italy) and then shipped to the Guided Therapeutics (GTx) Laboratory (University Health Network, University of Toronto, Toronto, ON, Canada).

### Human bone marrow mesenchymal stromal cell (hMSCs) culture

Bone marrow hMSCs (BM-hMSCs) were harvested, isolated, and expanded to passage 3 or 4 (P3-4) before being used for the study; BM-hMSCs were donated from healthy consenting donors under an approved protocol in the Viswanathan Lab (Krembil Research Institute, University Health Network, University of Toronto, Toronto, ON, Canada). For hMSC expansion, 5% human platelet lysate (hPL, Stemcell Technologies), Dulbecco’s Modified Eagle Medium (DMEM, Sigma Aldrich), a high glucose-based medium with 2% L glutamine/penicillin-streptomycin/amphotericin B solution (stock solution, 200 mM L-glutamine, 10,000 U/mL penicillin, 10 mg/mL streptomycin, 250 μg/mL amphotericin B), 1 mM sodium pyruvate, and MEM non-essential amino acids solution (1X) were employed.

### *In vitro* and *in vivo* phases

Scaffolds were immersed in analogous growth medium seeded with hMSCs at different concentrations (1,000, 2,000, and 3,000 cells/mm^3^ of the scaffold volume); this was considered as time 0. The growth medium was renewed every 24 h under sterile conditions. On day 4 (*i.e.*, 72 h after seeding of scaffolds), the scaffolds were randomly divided into two groups (1:1 ratio), each undergoing a different experimental procedure, as follows.

1) *in vitro* cell viability assay: scaffolds were removed from the growth medium, stained with calcein (Invitrogen–Thermo Fisher Scientific; green, live cells) and propidium iodide (Bioshop; red, dead cells) following the manufacturer’s instructions, and subsequently scanned with a 2-channel epifluorescence microscope (red, green) (AxioZoom microscope [Zeiss] with Plan NeoFluar Z ×1 objective NA 0.25 and, an X-Cite 120 metal halide lamp). Images were acquired using a Hamamatsu ORCA Flash v2 sCMOS camera. Subsequently, images were deconvolved using Huygens Professional (Scientific Volume Imaging), and analyzed using Imaris (Bitplane Software, a Division of Oxford Imaging). This experiment aimed to demonstrate the presence of viable hMSCs in scaffolds at the time of surgery.
2) mandibular implantation in a rabbit model: 17 rabbits were used for the experimental study. Of these, 1 (5.9%) died in the early postoperative period (postoperative day [POD] 19), and thus 16 animals composed the study sample for measurements reported below. Overall, 24 surgical defects were realized and 21 scaffolds implanted. Table 1 and Supplementary Table S1 summarize surgical site distribution among study subgroups and subgroup clustering, respectively.

**TABLE 1:** Distribution of experimental reconstruction strategies employed in the study.

Study population	Site of the defect	Size of defect	Reconstruction strategy
16 rabbits (24 surgical implants)	Cervical (20)	Small (16) 5 × 3 × 3mm^3^	No reconstruction (3)^C1^ HyCh (1)^C2^ PLA-PCL-HyCh (2)^C2^ HyCh + 1K-hMSCs (1) PLA-PCL-HyCh + 1K-hMSCs (1) HyCh + 2K-hMSCs (2) PLA-PCL-HyCh + 2K-hMSCs (2) HyCh + 3K-hMSCs (2) PLA-PCL-HyCh + 3K-hMSCs (2)
		Large (4) 15 × 3 × 3mm^3^	HyCh + 2K-hMSCs (1) PLA-PCL-HyCh + 2K-hMSCs (1) HyCh + 3K-hMSCs (1) PLA-PCL-HyCh + 3K-hMSCs (1)
	Oral (4)	Small (4) 3 × 3 × 3 mm^3^	HyCh + 2K-hMSCs (1) PLA-PCL-HyCh + 2K-hMSCs (1) HyCh + 3K-hMSCs (1) PLA-PCL-HyCh + 3K-hMSCs (1)

Numbers in round parentheses refer to the number of surgical defects. C1, Controls with no reconstruction; C2, controls with unseeded scaffold-based reconstruction.

### Primary surgery

Three different types of surgeries were performed on rabbits under general anesthesia with inhalant isoflurane (induction: 4 L/min; maintenance 1.5 L/min), after perioperative medication with antibiotic prophylaxis (intravenous cefazoline, 20 mg/kg) and analgesia (subcutaneous buprenorphine, 0.05 mg/kg) 30 min before surgery.

**FIGURE 3:**
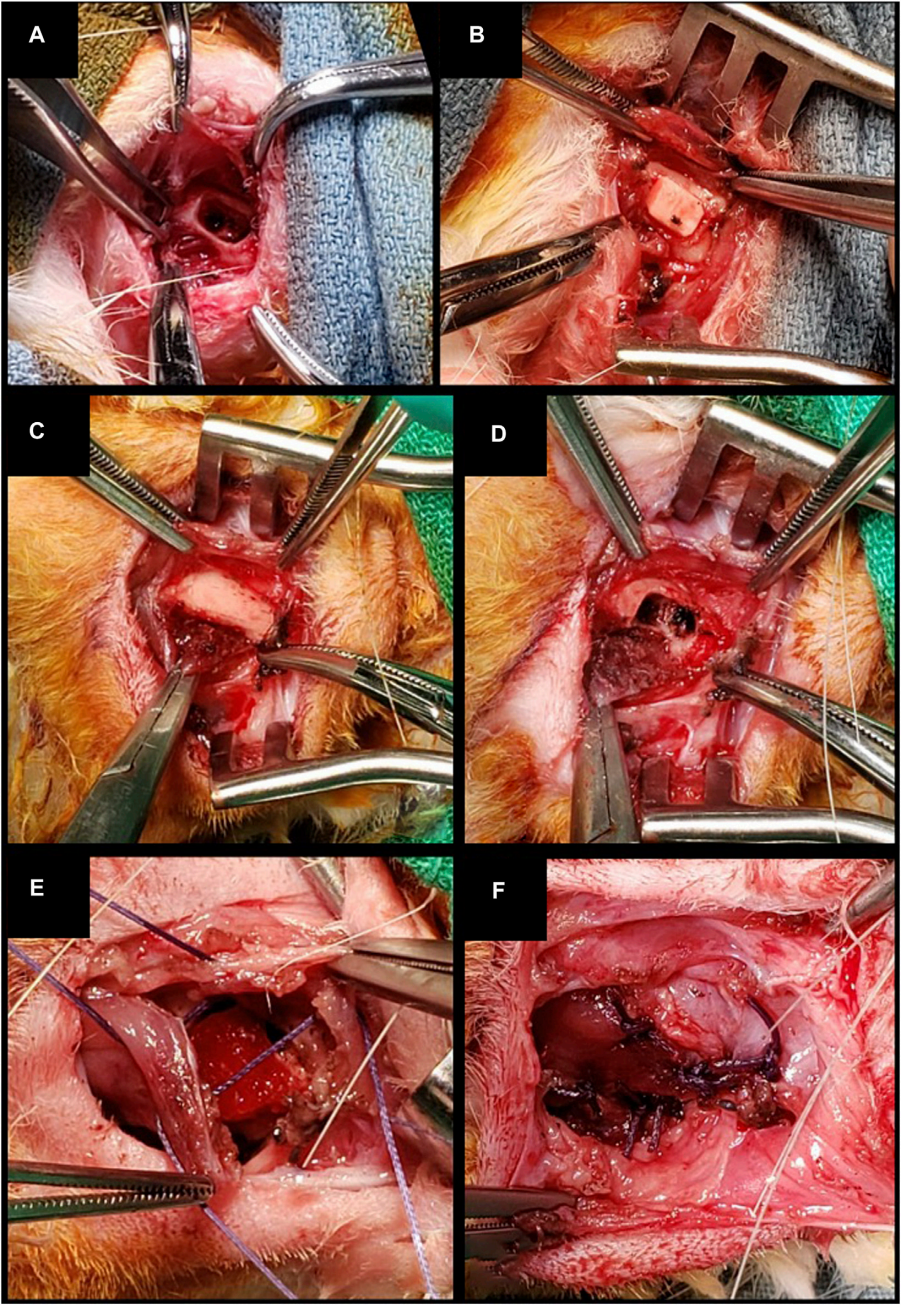
**(A, B)** Inferior marginal mandibulectomy and positioning of a scaffold made of PLA-PCL-HyCh. **(C–F)** Inferior marginal mandibulectomy and positioning of a scaffold made of HyCh, secured by suturing adjacent soft tissues.

**FIGURE 4:**
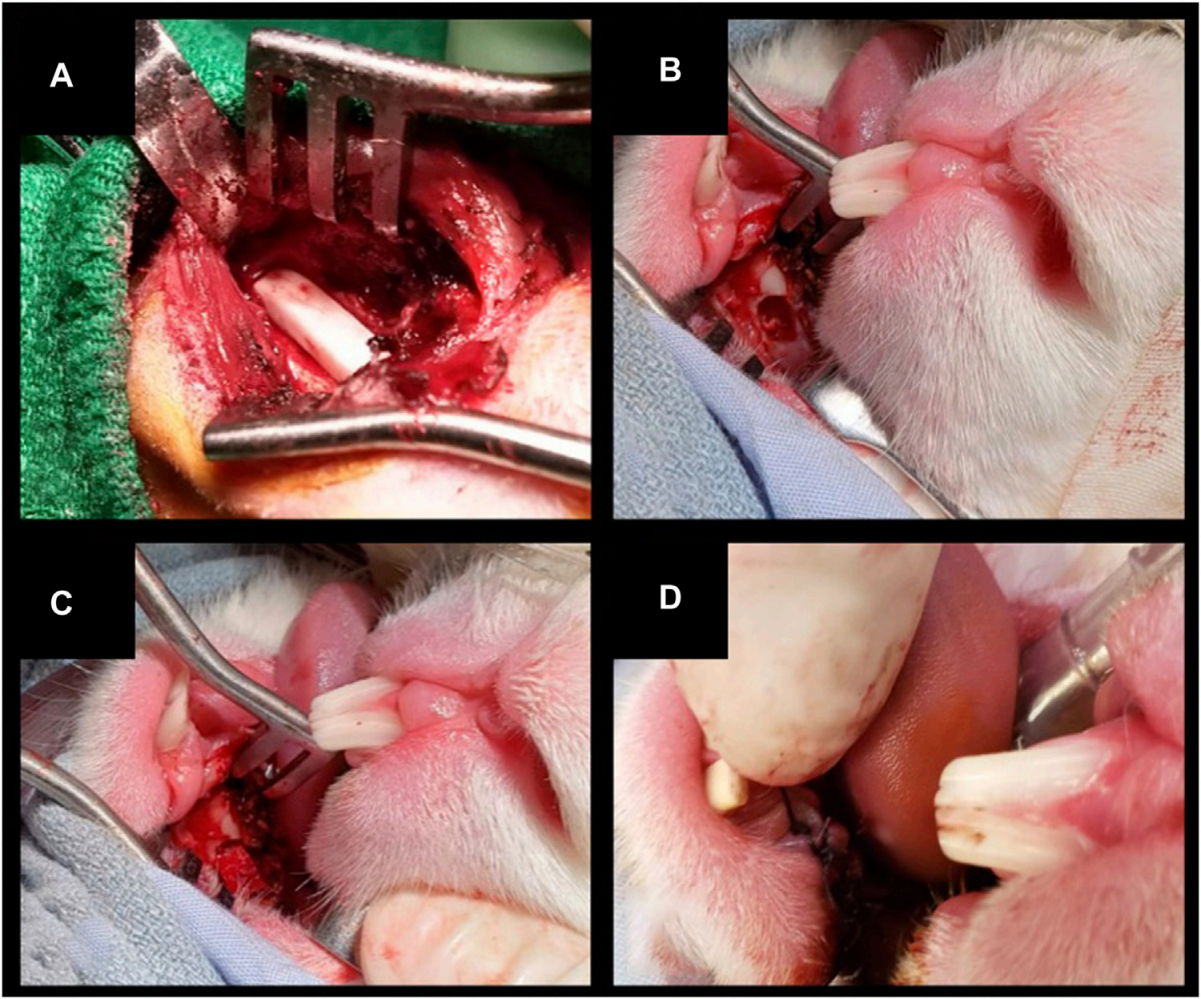
**(A)** Inferior marginal mandibulectomy to create a large defect (15 × 3 × 3 mm^3^) and positioning of a scaffold made of PLA-PCL-HyCh. **(B–D)** Transoral teeth-sparing superior marginal mandibulectomy and positioning of a scaffold made of HyCh, secured by suturing the adjacent oral mucosa.

1) Bilateral inferior mandibulectomy (small defect): the inferior border of the mandible was exposed bilaterally through a 2-cm incision along the midline of the suprahyoid area. Periosteum and muscular insertions were dissected off the inferior aspect of the mandibular body and removed. Defects measuring 5 × 3 × 3 mm^3^ (with 5 mm set along the greatest axis of the mandibular body) were drilled out at the inferior border of the mandible. After cauterizing the edges of the defect, scaffolds were positioned and secured by suturing a cuff of neighboring soft tissues. In control animals, bony defects were either filled with an unseeded scaffold or left unreconstructed. This procedure was performed on 8 animals (Figure 3).
2) Unilateral inferior mandibulectomy (large defect): the inferior border of the mandible was exposed unilaterally through a 2-cm incision along the midline of the suprahyoid area. Defects measuring 15 × 3 × 3 mm^3^ (with 15 mm set along the greatest axis of the mandibular body) were drilled out at the inferior border of the mandible. The scaffold was positioned and secured by suturing a cuff of neighboring soft tissues. Large defects were created unilaterally since bilateral surgery with no mechanical stabilization was thought to increase unacceptably the risk of pathologic fractures. This procedure was performed on 4 animals (Figure 4A).
3) Unilateral transoral teeth-sparing mandibulectomy (transoral defect): a horizontal, 1 cm long incision was made in the oral mucosa located between incisors and molars on one side. The mental nerve was identified and divided, and the respective bony foramen drilled to create a defect measuring 3 × 3 × 3 mm^3^ at the superior border of mandible. After removing the adjacent periosteum, the scaffold was positioned and secured by suturing a cuff of neighboring soft tissues. Transoral defects were created unilaterally since bilateral surgery was thought to increase unacceptably the risk of orocervical fistulization, osteitis/osteomyelitis, and neck infection. This procedure was performed on 4 animals (Figures 4B–D).

### Animal monitoring and adverse events assessment and management

After surgery, animals were submitted to a daily clinical veterinary control, including evaluation of overall status, activity, feeding capacity, signs of pain, surgical wound status, urinary and fecal output, and body temperature. Weight was evaluated weekly, while biochemical monitoring with complete blood count (CBC) and basic biochemistry (renal and liver function) was performed every 2 weeks. For the first 2 weeks after surgery, soft food with appetizers was administrated to avoid excessive mechanical solicitation of the mandible.

According to the animal use protocol, in case of severe adverse events detected by the veterinary team, the animal might reach a humane endpoint, prompting the need of euthanasia. Humane endpoints were defined in case of persistent abnormal posture, untreatable anorexia and dehydration, persistent self-trauma, hemorrhagic discharge, and surgical site alterations compromising normal behavior, or causing dysphagia.

### *In vivo* imaging acquisition and analysis

All rabbits underwent a CT scan (eXplore Locus Ultra MicroCT [General Electric, London, ON, Canada; voltage: 80 kV, current: 50 mA, isotropic voxel Size: 154 μm]) of the head and neck region before surgery. A biweekly radiological *in vivo* postoperative evaluation was also performed with the same scanner with and without contrast agent (Omnipaque iodine contrast agent [GE Healthcare, Chicago, IL, USA]). Imaging was acquired under general anesthesia with inhalant isoflurane (1.5 L/min).

**FIGURE 5:**
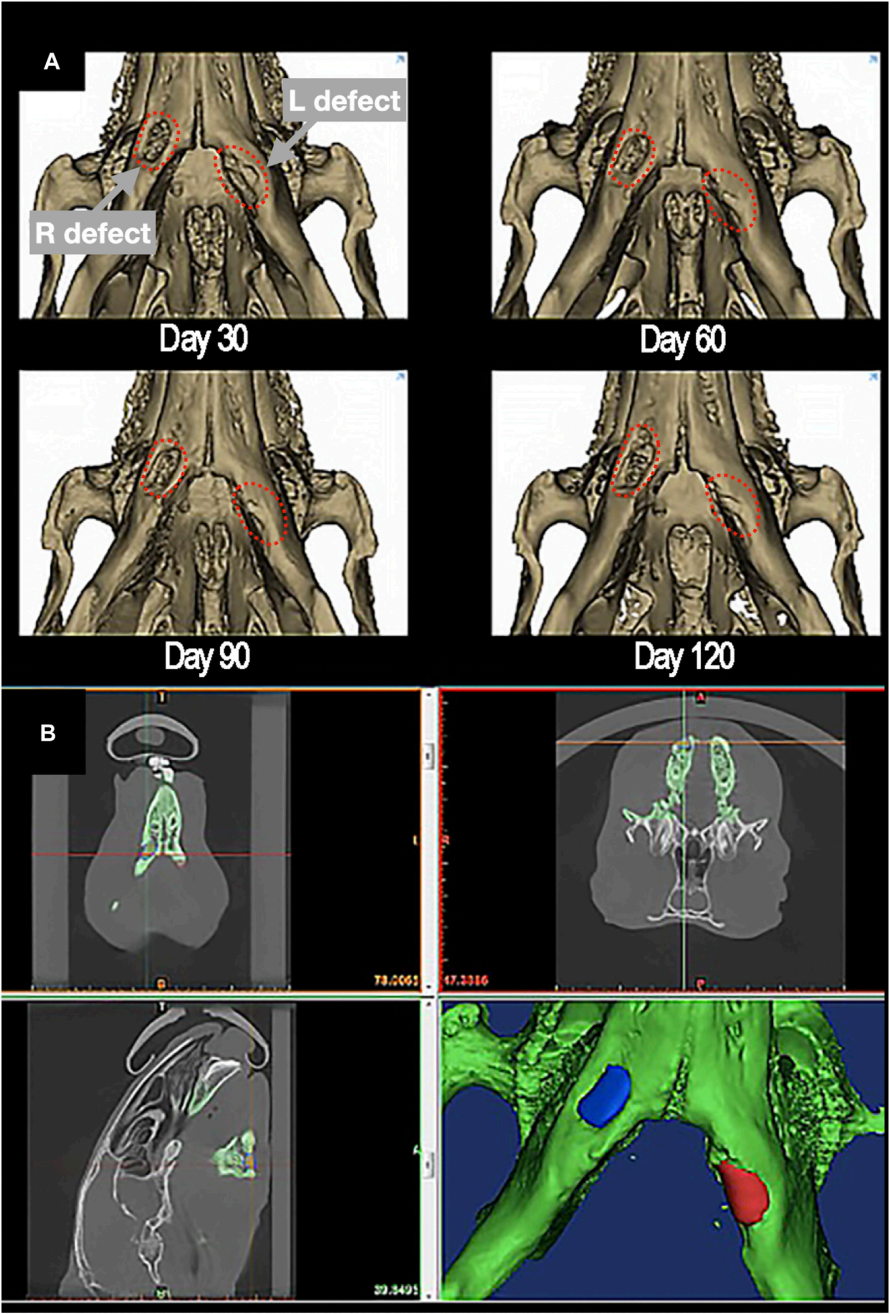
**(A)** 3D-rendering of postoperative restoration of a bilateral defect of inferior marginal mandibulectomy (red dashed-lines), reconstructed with scaffolds made of PLA-PCL-HyCh on the right side and HyCh on the left side, at different timepoints. **(B)** Methodology of measurement of the absolute density of the scaffolding area, identified and segmented in the first postoperative imaging.

The radiological images obtained were uploaded to 3D-modelling software (Mimics^®^/3-matic^®^ Materialise^®^; research software license; Leuven, Belgium). The surgical site was identified and segmented in the first postoperative imaging. To ensure topographic consistency throughout measurements, each CT was co-registered to the first postoperative mandible and defect rendering. The average density at the implant site was measured in Hounsfield Units (HU) in the non-contrast-enhanced (CE) acquisition. This value was defined as “absolute density”. The preoperative density at the implant site was considered as the complete restoration value (*i.e.*, 100% density restoration), while the first postoperative value acquired within 7–10 days after surgery was approximately defined as the baseline value (*i.e.*, 0% bone restoration). Thus, all absolute density measurements were rescaled and expressed as percentage, referred to as “relative density” (Figure 5).

**FIGURE 6:**
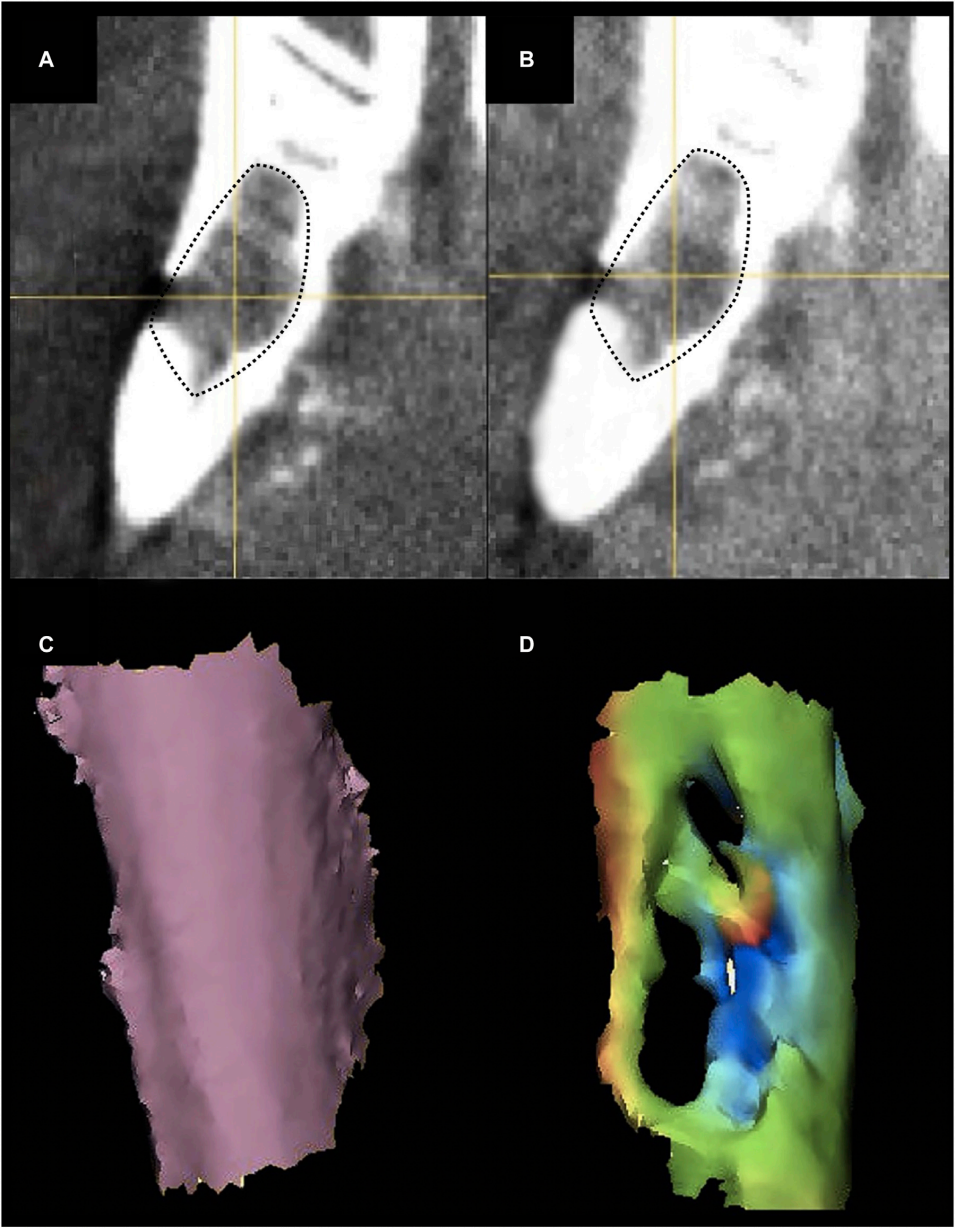
**(A, B)** Pre- **(A)** and post-contrast **(B)** agent injection CT scan of the mandibular defect. Contrast enhancement can be appreciated in the defect area (black dotted-line). **(C, D)** Example of preoperative cortical shape **(C)** and 30-day postsurgical cortical shape **(D)** of the defect area. A color-scale map quantifies the morphological similarity between postsurgical and presurgical shapes (green areas are similar to the original shape, orange-to-red areas are excessively protruding with respect to the original shape, blue areas are depressed with respect to the preoperative shape).

The uptake of contrast medium at the surgical site, referred to as “uptake”, was measured as the difference between the average density in the CE acquisition minus the average density in the non-CE acquisition (Figures 6A, B).

The external surface of the defect (*i.e.*, the bone surface in contact with soft tissue) was segmented from the preoperative and postoperative CTs. A part-comparison-analysis between each postoperative segmentation and the respective preoperative one was performed (Chan et al., 2010; Pagedar et al., 2012a; Chan et al., 2015; Davies et al., 2018). Root mean square (RMS) of the part-comparison-analysis output was registered and used as an estimate of morphological similarity of the postoperative segmentations with respect to the preoperative one (*i.e.*, low root mean square indicates high morphological similarity) (Figures 6C, D).

### Surgical endpoint

The scientific endpoint was set between 114 and 150 days from the surgical procedure. When the scientific endpoint was achieved, the animal was euthanized with an injection of 2.5 mL of potassium chloride (KCl) under general anesthesia obtained with inhalant isoflurane at 5% dosage. The mandible was then carefully removed, keeping the implant site protected and surrounded by a cuff of adjacent soft tissues.

### *Ex vivo* imaging

**FIGURE 7:**
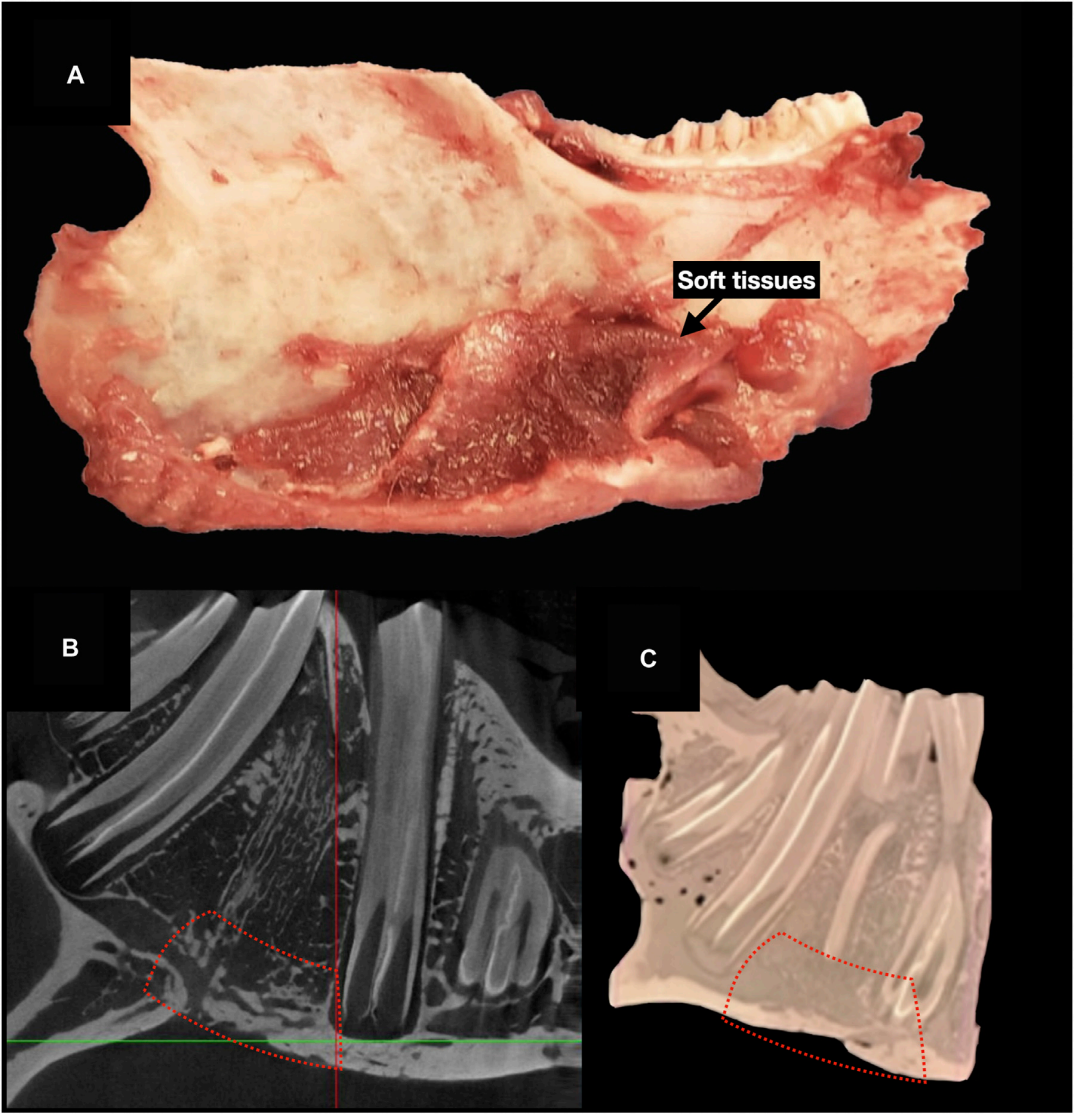
Ultra-high-definition CT on *ex vivo* specimens. **(A)**, The mandible is harvested after euthanasia. **(B)**, Cross-sectional 2D images on ultra-high-definition CT. **(C)**, Three-dimensional image reconstruction. Red dashed-line indicates the area of bone regeneration **(B, D)**.

The *ex vivo* radiological evaluation of the harvested specimens was performed by ultra-high-definition CT (SkyScan 1,276 microCT system [Bruker, Belgium; voltage: 85 kV, current: 47 μA, isotropic voxel size: 10 μm]). On the images obtained, a region of interest (ROI) corresponding to the surgical defect repaired with the scaffold was manually identified through comparison with the first postoperative imaging (Figure 7). The software CT Analyser 1.17.7.2 (Bruker^®^) was used to extract quantitative data regarding the ROI (hereby referred to as “microarchitectural bone characteristics”), namely: 1) bone volume as percentage of the overall tissue volume; 2) mean trabecular thickness; 3) trabecular density per mm^3^; and 4) mean trabecular separation in mm.

Sixteen ROIs were similarly analyzed in not-operated mandibles of 4 rabbits (bilaterally, n = 8) not included in the present study and of 8 rabbits included in the present study and receiving unilateral surgery (n = 8). The data extracted from this sample were used as an estimate of native bone microarchitectural bone characteristics.

### Specimen processing, staining, and histological imaging analysis

**FIGURE 8:**
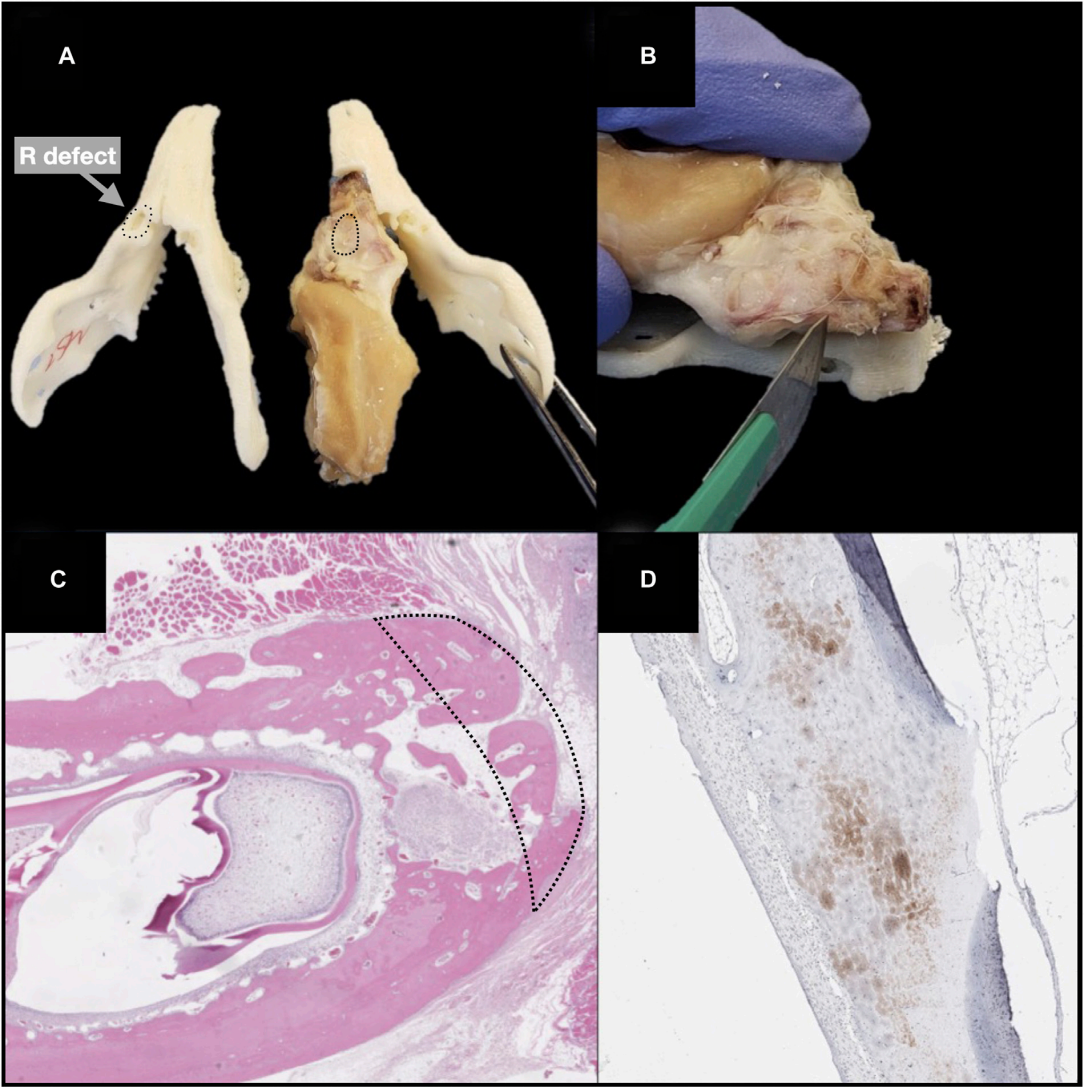
Steps of *ex vivo* specimen processing: production of a 3D printed model of each rabbit’s mandible, based on the first postoperative CT, and checking the morphological fitting with the decalcified *ex vivo specimen*; matching the surgically treated area of the specimen (black dashed line) with the corresponding site on the model (black dotted line) **(A)** cutting the specimen at the midpoint of the surgical defect area, to obtain the samples for histological analysis **(B)**; hematoxylin-eosin staining (black dashed line indicates the area of bone regeneration) **(D)**; immunohistochemical staining (anti-HNA) **(D)**.

The surgical specimen, including the mandible and soft tissue surrounding the implanted sites, underwent a decalcification process with ethylenediaminetetraacetic acid (EDTA). Before paraffin embedding, each sample was cut at the level of scaffold’s midpoint, obtaining two specimens to be subsequently processed with paracoronal histological slices (*i.e.*, with the cutting plane perpendicular to the greatest axis of the mandibular body). The site of the scaffold was identified by 3D-printing an actual-size mandibular model obtained from the first post-operative CT of each rabbit, thus comparing it to the harvested *ex vivo* mandibular specimen (3D Printer Dimension 1200es System Stratasys (Eden Prairie, MN, USA) (Figures 8A, B).

Histological sections were deparaffinized in xylene, rehydrated, and stained with H&E (Bio-Optica), to analyze general tissue morphology, and TRAP staining (Sigma-Aldrich, St. Louis, USA) to evaluate osteoclast activity, following the manufacturer’s staining protocols. Histological slices underwent immunohistochemical staining with anti-VEGF-A (mouse monoclonal [VG-1], Abcam, Cambridge, UK; dilution: 1:500), anti-bone sialoprotein (mouse monoclonal [ID1.2], Immundiagnostik, Bensheim, Germany; dilution: 1:600), anti-osteocalcin (mouse monoclonal [OCG3], Genetex, Irvine, USA; dilution: 1:200), anti-osteopontin (mouse monoclonal [1B20], Novus Biologicals, Littleton, USA; dilution: 1:200), anti-human nuclear antigen antibodies (mouse monoclonal [235–1], Abcam, Cambridge, UK; dilution: 1:800) (Figures 8C, D).

The slides were digitalized with an Aperio AT2 brightfield scanner (Leica Biosystems, Concord, ON, Canada) and expression of the immunohistochemical markers within each considered ROI was quantitatively evaluated in terms of percentage of stain-positive area over total tissue area, using an image analysis platform for quantitative tissue analysis in digital pathology (Halo [Indica Lab, Albuquerque, NM, US]). The ROI was defined as the surface occupied by bony tissue in each slide, accounting for the area of the surgical defect. These data are referred to as “histological bone characteristics”.

### Statistical analysis

Statistical analysis was performed using RStudio (Version 1.2.5042). Two types of data were gathered for analysis: 1) time-dependent data and 2) endpoint data. The first cluster included relative density, uptake, and conformance restoration, whereas the second entailed microarchitectural bone characteristics and histological bone characteristics. These data were considered as the response variables and association thereof with the following explanatory variables was checked: scaffold employment (yes *vs* no), scaffold seeding (yes *vs* no *vs* no reconstruction), hMSC seeding concentration (1,000 cells/mm^3^ *vs* 2,000 cells/mm^3^ *vs* 3,000 cells/mm^3^ *vs* controls), defect site (oral *vs* cervical *vs* controls), defect size (small, including both 3 × 3 × 3 mm^3^ and 5 × 3 × 3 mm^3^ defects, *vs* large *vs* controls), material (HyCh *vs* PLA-PCL-HyCh *vs* no reconstruction), material and seeding status (unseeded HyCh *vs* HyCh + hMSC *vs* unseeded PLA-PCL-HyCh *vs* PLA-PCL-HyCh + hMSC *vs* no reconstruction).

Time-dependent data were modelled as linear models and graphically rendered through generalized additive model-generated regression lines on scatter plots. Time-dependent values were estimated through linear regression models at 60- and 120-day timepoints. Comparison between explanatory variable-determined subgroups was performed through analysis of variance with estimated marginal mean-based Tukey-adjusted *post hoc* test. For endpoint data, observations outlying the time interval between 120 and 150 days after surgery were considered as non-consistently comparable with other observations and were thus ignored (n = 2: one animal was euthanized earlier than planned [POD 106] for COVID-19-pandemic-related logistical constraints; another animal was euthanized earlier than planned due to reaching a humane endpoint owing to pulmonary atelectasis [POD 71]). Endpoint data were graphically rendered through violin plots and analyzed through the Mann-Whitney test (for dichotomous explanatory variables) and the Kruskal-Wallis test (for non-dichotomous explanatory variables). Significance was set at 0.05 for all statistical tests. *p*-values comprised between 0.05 (included) and 0.10 (excluded) were considered “close-to-significance”.

### Ethics

The protocols (AUP#6010; title: Primary reconstruction of maxillary and mandibular defects with computer-aided designing, computer-aided manufacturing bioengineered composite scaffolds) for experimentation on animals were approved by the University Health Network Animal Care Committee (Princess Margaret Cancer Centre, University Health Network, University of Toronto) in April 2019. All authors confirm their compliance with all relevant ethical regulations.

## Results

### *In vitro* viability assay of bioengineered scaffolds

**FIGURE 9:**
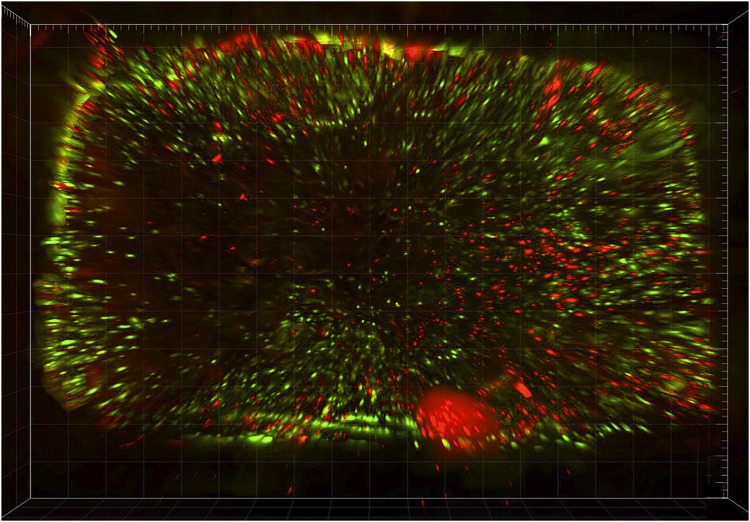
*In vitro* cell viability assay to assess and quantify the presence of vital cells: 3-dimensional rendering of an epifluorescence microscopy scanning of a scaffold seeded with human mesenchymal stromal cells and stained with calcein and propidium iodide, which mark living and dead cells in green and red, respectively.

All (100%) randomly selected scaffolds showed viable cells (Figure 9) at the time of surgery (*i.e.* 72 h after seeding of scaffolds). Mean cellular viability (viable cells/total cells) resulted 49.1% (range: 42.3%–56.7%), and mean viable cells density 234 (range: 198–327) viable cells/mm^3^.

### *In vivo* regenerative performance of bioengineered scaffolds

**FIGURE 10:**
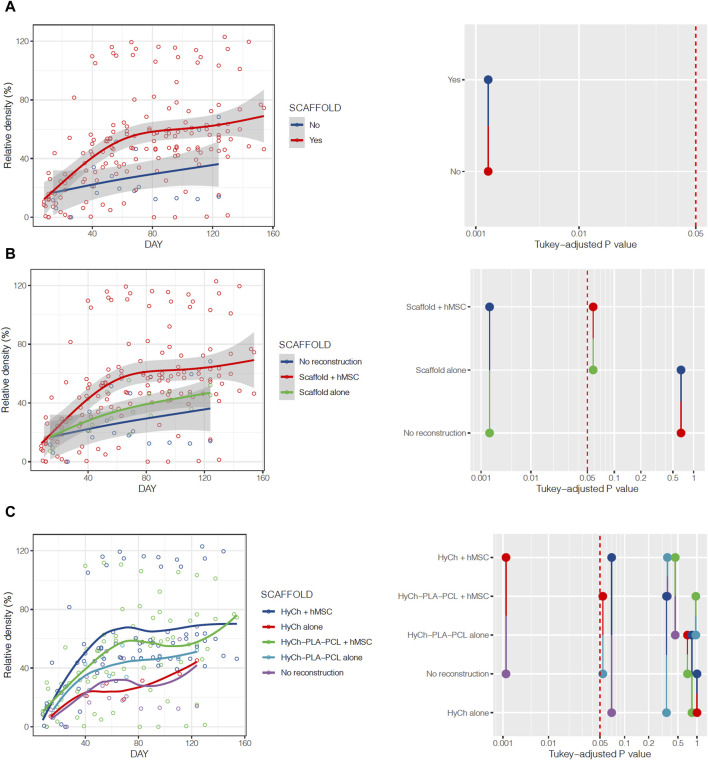
Relative density of the surgical site over time, stratified by presence or absence of the scaffold **(A)**, employment of seeded vs. unseeded scaffold **(B)**, and according to seeding status and material composing the scaffold **(C)**. Pairwise comparisons between categories and relative *p*-values are represented on the right of the figure.

All animals showed a spontaneous trend of relative density increase (RDI) over time at the surgical site. RDI was significantly more pronounced in defects where a scaffold was placed as opposed to non-reconstructed sites (*p* = 0.0018), particularly for scaffolds seeded with hMSCs (*vs* non-reconstructed sites *p* = 0.0018; unseeded scaffolds *vs* non-reconstructed sites *p* = 0.6459) (Figure 10; Table 2). Overall, HyCh and PLA-PCL-HyCh did not show a significantly different RDI (*p* = 0.2693), with both outperforming controls (*p* = 0.0014 and *p* = 0.0255, respectively). When considering the seeding status, seeded HyCh scaffolds showed the best performance in terms of RDI and they were the only subgroup with a statistically significant difference compared to non-reconstructed sites (*p* = 0.0013). RDI of seeded PLA-PCL-HyCh scaffolds were close-to-significantly higher than that of non-reconstructed sites (*p* = 0.0541).

**TABLE 2:** Estimates of relative density (RD) at 60 and 120 days after surgery, clustered by several explanatory variables considered in the study.

Clustering variable	60-day RD (%)	120-day RD (%)	*p*-value*
None (entire series)	42.7	64.6	N.A.
Scaffold (no *vs* yes)	No: 25.0 Yes: 44.8	No: 40.1 Yes: 66.9	**0.0018**
Scaffold type (no recon. *vs* HyCh *vs* PLA-PCL-HyCh)	No recon.: 25.0 HyCh: 47.8 PLA-PCL-HyCh: 42.0	No recon.: 40.1 HyCh: 71.5 PLA-PCL-HyCh: 62.3	**0.0023**
Scaffold seeding status (no recon. *vs* seeded scaffold *vs* unseeded scaffold)	No recon.: 25.0 Seeded scaffold: 46.4 Unseeded scaffold: 32.4	No recon.: 40.1 Seeded scaffold: 68.7 Unseeded scaffold: 49.2	**0.0006**
Scaffold type and seeding status (no recon. *vs* HyCh ± hMSCs *vs* PLA-PCL-HyCh ± hMSCs)	No recon.: 25.0 HyCh alone: 23.7 HyCh + hMSCs: 50.2 PLA-PCL-HyCh alone: 37.4 PLA-PCL-HyCh + hMSCs: 42.7	No recon.: 40.1 HyCh alone: 41.6 HyCh + hMSCs: 74.1 PLA-PCL-HyCh: 54.9 PLA-PCL-HyCh + hMSCs: 63.2	**0.0007**
Defect site (no seeding/no scaffold *vs* cervical *vs* oral)	No seeding/no scaffold: 28.7 Cervical: 43.7 Oral: 56.8	No seeding/no scaffold: 44.6 Cervical: 64.3 Oral: 86.2	**<0.0001**
Defect size (no seeding/no scaffold *vs* small *vs* large)	No seeding/no scaffold: 28.7 Small: 47.3 Large: 44.0	No seeding/no scaffold: 44.6 Small: 71.9 Large: 60.8	**0.0005**
hMSCs concentration (no cells *vs* 1,000 cells/mm^3^ *vs* 2,000 cells/mm^3^ *vs* 3,000 cells/mm^3^)	No seeding/no scaffold: 28.7 1,000 cells/mm^3^: 41.4 2,000 cells/mm^3^: 45.1 3,000 cells/mm^3^: 49.4	No seeding/no scaffold: 44.6 1,000 cells/mm^3^: 61.9 2,000 cells/mm^3^: 62.0 3,000 cells/mm^3^: 78.1	**0.0006**

*The *p*-value refers to the analysis of variance test (ANOVA) on linear regression models, see the text for relevant *post hoc* pairwise comparisons between categories; significant *p*-values are reported as bold. hMSC, human mesenchymal stromal cell; HyCh, hydrogel-chitosan scaffolds; PLA-PCL-HyCh, polylactic acid-polycaprolactone-hydrogel chitosan scaffolds.

Despite no statistical significance difference (*p* = 0.1212) was observed between the two groups, non-reconstructed sites showed higher initial uptake in comparison to scaffold-including sites. Moreover, the former group showed a decreasing trend in uptake over time, whereas the latter group displayed a stable-to-mildly-increasing uptake over time. Addition of hMSCs to scaffolds created a small decrease in uptake, although with no significant difference (*p* = 0.2930) (Supplementary Table S2). Sites implanted with HyCh scaffolds as well as those with no reconstruction were significantly more permeable to the contrast agent than those with PLA-PCL-HyCh (*p* = 0.0019 and *p* = 0.0309, respectively). Both HyCh and PLA-PCL-HyCh scaffolds had a more stable uptake value over time when seeded with hMSCs, whereas controls showed a more variable trend.

Reduction of root mean square at part-comparison-analysis (RRP), which measures the similarity of the cortical bony contour of the surgical site compared to the preoperative shape, was greater in defects reconstructed with a scaffold, although with no statistical significance (*p* = 0.7665) (Supplementary Table S3).

### *Ex vivo* regenerative performance of bioengineered scaffolds

**FIGURE 11:**
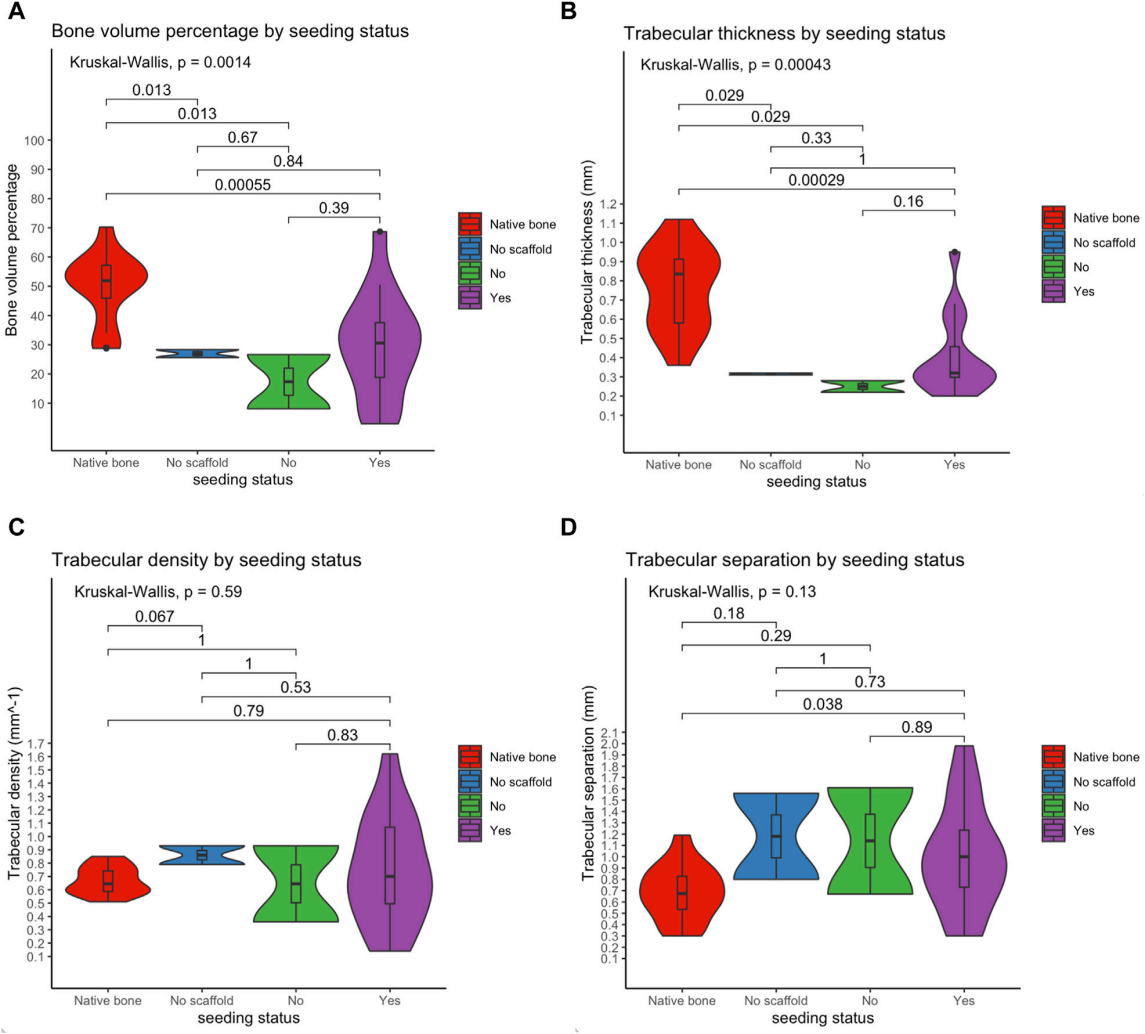
Violin plots showing that the microarchitectural characteristics of regenerated bone did not equate those of the native bone, irrespective of the employment of a scaffold and seeding with human mesenchymal stromal cells. Of note, among study subgroups only the seeded scaffold included some cases equating to native bone in terms of bone volume and trabecular thickness. Bone volume percentage **(A)**; trabecular thickness **(B)**; trabecular density **(C)**; trabecular separation **(D)**.

Microarchitectural bone characteristics and their association with explanatory variables are summarized in Table 3. Native bone characteristics were significantly better (*i.e.*, higher relative bone volume, higher trabecular density, higher trabecular thickness, and lower intertrabecular distance) than regenerated bone, regardless the presence of a scaffold in the surgical site and seeding status (Figure 11).

**TABLE 3:** Microarchitectural bone characteristics, clustered by several explanatory variables considered in the study.

Clustering variable	Median percentage of bone (%)	Median trabecular thickness (mm)	Median trabecular density (mm^-1^)	Median trabecular separation (mm)
None (entire series)	36.7	0.53	0.70	0.81
Scaffold	NB: 51.9 No: 27.0 Yes: 27.4 **p = 0.0006**	NB: 0.84 No: 0.32 Yes: 0.30 **p = 0.0002**	NB: 0.65 No: 0.86 Yes: 0.70 *p* = 0.3907	NB: 0.68 No: 1.18 Yes: 1.00 *p* = 0.0597
Scaffold type	NB: 51.9 NR: 27.0 Hy: 33.3 P: 19.3 **p = 0.0017**	NB: 0.84 NR: 0.32 Hy: 0.31 P: 0.30 **p = 0.0007**	NB: 0.65 NR: 0.86 Hy: 0.90 P: 0.51 *p* = 0.0934	NB: 0.68 NR: 1.18 Hy: 0.76 P: 1.20 *p* = 0.0797
Scaffold seeding status	NB: 51.9 NR: 27.0 Seeded: 31.0 Unseeded: 17.4 **p = 0.0014**	NB: 0.84 NR: 0.32 Seeded: 0.32 Unseeded: 0.25 **p = 0.0004**	NB: 0.65 NR: 0.86 Seeded: 0.70 Unseeded: 0.65 *p* = 0.5897	NB: 0.68 NR: 1.18 Seeded: 1.00 Unseeded: 1.14 *p* = 0.1284
Scaffold type and seeding status	NB: 51.9 NR: 27.0 Hy: 26.6 Hy + hMSCs: 33.9 P-Hy: 8.1 P-Hy + hMSCs: 21.4 **p = 0.0069**	NB: 0.84 NR: 0.32 Hy: 0.28 Hy + hMSCs: 0.35 P-Hy: 0.22 P-Hy + hMSCs: 0.32 **p = 0.0026**	NB: 0.65 NR: 0.86 Hy: 0.93 Hy + hMSCs: 0.82 P-Hy: 0.36 P-Hy + hMSCs: 0.58 *p* = 0.1716	NB: 0.68 NR: 1.18 Hy: 0.67 Hy + hMSCs: 0.79 P-Hy: 1.61 P-Hy + hMSCs: 1.11 *p* = 0.1486
Defect site	NB: 51.9 NSNS: 26.1 Cervical: 30.6 Oral: 30.7 **p = 0.0014**	NB: 0.84 NSNS: 0.30 Cervical: 0.32 Oral: 0.28 **p = 0.0005**	NB: 0.65 NSNS: 0.86 Cervical: 0.70 Oral: 0.81 *p* = 0.8315	NB: 0.68 NSNS: 1.18 Cervical: 1.02 Oral: 0.71 *p* = 0.0798
Defect size	NB: 51.9 NSNS: 26.1 Small: 35.6 Large: 18.4 **p = 0.0004**	NB: 0.84 NSNS: 0.30 Small: 0.34 Large: 0.30 **p = 0.0004**	NB: 0.65 NSNS: 0.86 Small: 0.72 Large: 0.55 *p* = 0.4783	NB: 0.68 NSNS: 1.18 Small: 0.79 Large: 1.46 **p = 0.0146**
hMSCs concentration	NB: 51.9 NSNS: 26.1 1 K cells/mm^3^: 31.8 2 K cells/mm^3^: 32.3 3 K cells/mm^3^: 26.3 **p = 0.0028**	NB: 0.84 NSNS: 0.30 1 K cells/mm^3^: 0.45 2 K cells/mm^3^: 0.30 3 K cells/mm^3^: 0.33 **p = 0.0013**	NB: 0.65 NSNS: 0.86 1 K cells/mm^3^: 0.70 2 K cells/mm^3^: 0.82 3 K cells/mm^3^: 0.60 *p* = 0.8252	NB: 0.68 NSNS: 1.18 1 K cells/mm^3^: 1.00 2 K cells/mm^3^: 0.92 3 K cells/mm^3^: 0.98 *p* = 0.2024

*p*-values refer to the Kruskal-Wallis test. 0–3 K, 0/1,000/2,000/3,000 cells/mm^3^ at time of scaffold seeding; hMSC, human mesenchymal stromal cell; Hy, hydrogel-chitosan scaffolds; P, polylactic acid-polycaprolactone-hydrogel chitosan scaffolds; NB, native bone; NR, no reconstruction; NSNS, no seeding/no scaffold.

**FIGURE 12:**
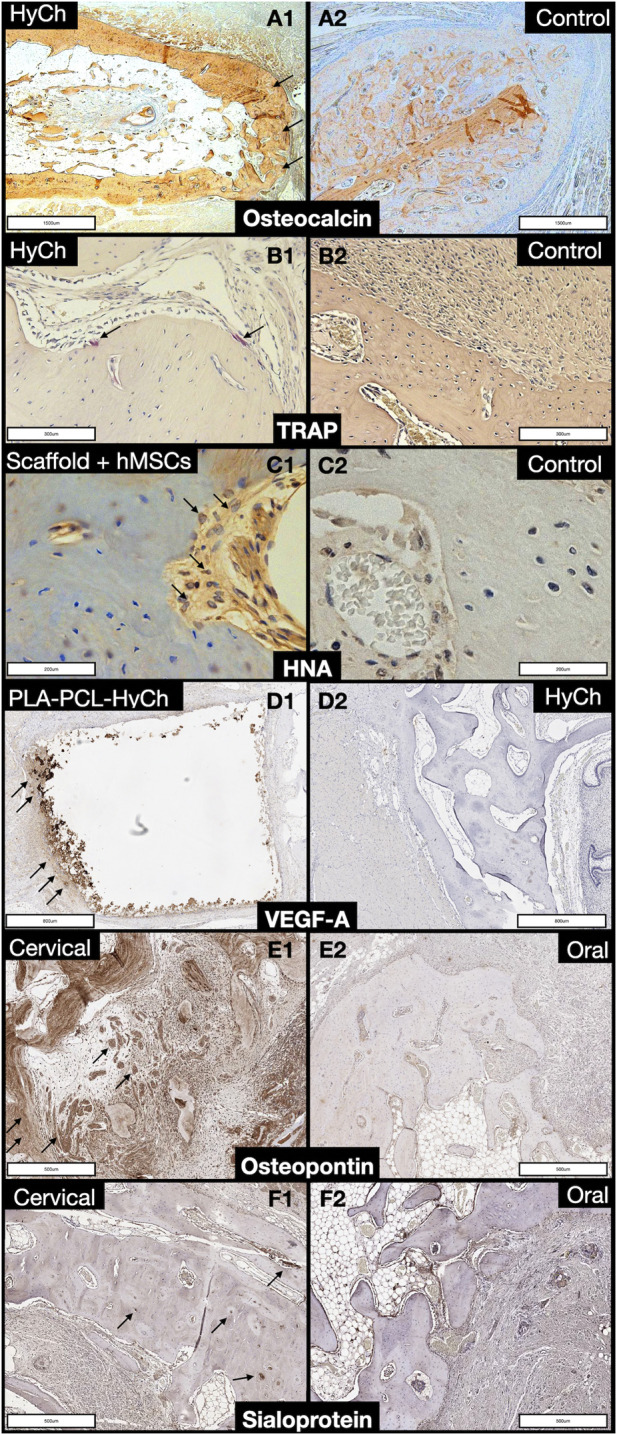
Panel displaying histochemical and immunohistochemical staining of regenerated bone. **A1, A2.** Comparison between a defect reconstructed with a HyCh scaffold (arrows) and one left unreconstructed (control) in terms of osteocalcin expression. **B1, B2.** Comparison between a defect reconstructed with a HyCh scaffold (arrows) and one left unreconstructed (control) in terms of tartrate-resistant acid phosphatase (TRAP) expression. **C1, C2.** Comparison between a defect reconstructed with a HyCh scaffold seeded with human mesenchymal stromal cells (hMSCs) and one left unreconstructed (control) in terms of human nuclear antigen (HNA) expression (arrows). **D1, D2.** Comparison between a defect reconstructed with a PLA-PCL-HyCh scaffold (arrows) and on reconstructed with HyCh in terms of vascular-endothelial growth factor-A (VEGF-A) expression. **E1, E2.** Comparison between a cervical defect (arrows) and an oral defect in terms of osteopontin expression. **F1, F2.** Comparison between a cervical defect (arrows) and an oral defect in terms of sialoprotein expression.

On histomorphological analysis, all surgical sites showed mixed bone (*i.e.*, cortical and spongious). Use of a scaffold also affects the immunohistochemical profile of the regenerated bone (Table 4): 1) VEGF-A was significantly more expressed in defects reconstructed with a PLA-PCL-HyCh scaffold compared with HyCh ones and controls (*p* = 0.0123); 2) Osteopontin was significantly more expressed in defects reconstructed with a scaffold than those left unreconstructed (*p* = 0.0332) (Figure 12).

**TABLE 4:** Histological and immunohistochemical bone characteristics, clustered by explanatory variables considered in the study.

Clustering variable	HNA SA/TSA (%)	Osteocalcin SA/TSA (%)	Osteopontin SA/TSA (%)	Sialoprotein SA/TSA (%)	TRAP SA/TSA (%)	VEGF-A SA/TSA (%)
Scaffold	No: 0.37 Yes: 1.03 *p* = 0.0807	No: 22.50 Yes: 20.91 *p* = 0.9337	No: 0.67 Yes: 6.05 **p = 0.0332**	No: 0.98 Yes: 2.91 *p* = 0.4739	No: 0.34 Yes: 0.23 *p* = 0.8407	No: 0.21 Yes: 0.21 *p* = 0.9062
Scaffold type	NR: 0.37 Hy: 1.39 P: 0.43 *p* = 0.0709	NR: 22.95 Hy: 15.86 P: 22.70 *p* = 0.5063	NR: 0.67 Hy: 7.18 P: 4.65 *p* = 0.1004	NR: 0.98 Hy: 3.12 P: 2.77 *p* = 0.7536>	NR: 0.34 Hy: 0.38 P: 0.15 *p* = 0.4385	NR: 0.21 Hy: 0.14 P: 0.32 **p = 0.0123**
Scaffold seeding status	NR: 0.37 Seeded: 1.03 Unseeded: 0.76 *p* = 0.2110	NR: 22.50 Seeded: 18.751 Unseeded: 28.345 *p* = 0.6079	NR: 0.67 Seeded: 6.05 Unseeded: 7.03 *p* = 0.0937	NR: 0.98 Seeded: 3.06 Unseeded: 0.17 *p* = 0.2576	NR: 0.34 Seeded: 0.30 Unseeded: 0.20 *p* = 0.9787	NR: 0.21 Seeded: 0.20 Unseeded: 0.27 *p* = 0.7835
Defect site	NSNS: 0.37 Cervical: 1.03 Oral: 1.64 *p* = 0.1925	NSNS: 22.50 Cervical: 22.70 Oral: 9.71 **p = 0.0200**	NSNS: 0.67 Cervical: 9.83 Oral: 3.41 **p = 0.0124**	NSNS: 0.98 Cervical: 1.86 Oral: 4.08 **p = 0.0226**	NSNS: 0.34 Cervical: 0.23 Oral: 0.29 *p* = 0.6988	NSNS: 0.21 Cervical: 0.21 Oral: 0.22 *p* = 0.9557
Defect size	NSNS: 0.37 Small: 0.53 Large: 1.54 *p* = 0.1620	NSNS: 22.50 Small: 21.65 Large: 15.33 *p* = 0.5072	NSNS: 0.67 Small: 6.05 Large: 7.00 *p* = 0.0991	NSNS: 0.98 Small: 3.09 Large: 2.41 *p* = 0.7419	NSNS: 0.34% Small: 0.41 Large: 0.13 **p = 0.0295**	NSNS: 0.21 Small: 0.21 Large: 0.13 *p* = 0.4640
hMSCs concentration	0–1 K cells/mm^3^: 0.48 2–3K cells/mm^3^: 1.33 **p = 0.0433**	0–1 K cells/mm^3^: 22.60 2–3 K cells/mm^3^: 18.75 *p* = 0.6408	0–1 K cells/mm^3^: 4.80 2–3 K cells/mm^3^: 5.96 *p* = 0.4483	0–1 K cells/mm^3^: 0.91 2–3 K cells/mm^3^: 3.12 *p* = 0.1185	0–1 K cells/mm^3^:0.42 2–3 K cells/mm^3^: 0.22 *p* = 0.1904	0–1 K cells/mm^3^: 0.20 2–3 K cells/mm^3^: 0.21 *p* = 0.4990

*p*-values refer to the Kruskal-Wallis test. 0–3 K, 0/1,000/2,000/3,000 cells/mm^3^ concentration at time of scaffold seeding; hMSC, human mesenchymal stromal cell; Hy, hydrogel-chitosan scaffolds; P, polylactic acid-polycaprolactone-hydrogel chitosan scaffolds; NB, native bone; NR, no reconstruction; NSNS, no seeding/no scaffold; SA, stained area; TSA, total selected area.

### Effects of cell concentration at the time of seeding

Cell concentration at seeding also influenced RDI, with only 2,000 and 3,000 cells/mm^3^ being associated with significantly higher RDI compared to controls (*p* = 0.0144 and *p* = 0.0002, respectively). RRP over time was significantly associated with hMSC concentration at seeding. In fact, 2,000 and 3,000 cells/mm^3^ showed greater RRP than 1,000 cells/mm^3^ (*p* = 0.0005 and *p* = 0.0031, respectively). Human nuclear antigen was significantly more expressed in defects reconstructed through a scaffold seeded with 2,000 and 3,000 cells/mm^3^ compared with 1,000 cells/mm^3^ and no seeding (*p* = 0.0433).

### Effects of defect size and type

There was no significant difference in terms of RDI relative to the size of the defect (small *vs* large *p* = 0.6407), while both small and large defects showed higher RDI if reconstructed with seeded scaffolds in contrast to controls (*p* = 0.0002 and *p* = 0.0444, respectively). The size of the defect was associated with uptake trend over time, with only reconstructed large defects showing significantly lower uptake compared with controls (*p* = 0.0205). TRAP stain was significantly associated with defect size, with large defects showing a lower staining value compared with small defects (*p* = 0.0295).

Defects of the oral aspect of the mandible showed a higher RDI compared to those located on the cervical aspect (*p* = 0.0213). Both sites showed a higher RDI if reconstructed with seeded scaffolds in contrast to controls (*p* < 0.0001 and *p* = 0.0042, respectively). Uptake of defects of the oral aspect of the mandible was similar and higher compared with non-reconstructed (*p* = 0.8966) and reconstructed mandibular cervical sites (*p* = 0.0048), respectively. Defects of the oral aspect of the mandible were associated with greater RRP than those created through the neck (*p* < 0.0001). Bone sialoprotein was more expressed in defects of the oral aspect of the mandible (*p* = 0.0226), and osteocalcin and osteopontin in those of the cervical surface of the mandible (*p* = 0.0200 and *p* = 0.0124, respectively).

### Mortality and adverse events

Out of 17 rabbits initially included in the study, 1 died on POD 19, for a perioperative (*i.e.*, within 1 month) mortality of 5.9%. This animal developed an infectious pneumonia with atelectasis and was euthanized as the humane endpoint was deemed reached. Among the remaining 16 rabbits, one animal was found dead on POD 71 and the autopsy showed pulmonary hemorrhage, cardiomegaly, and coronary thrombosis. In neither of these two cases could a clear relationship with the experimental protocol be established.

All animals ate and showed regular urinary and fecal output within 48 h from surgery. Serial peripheral blood examination did not show any clinically relevant variations in terms of hemoglobin, cell count (*i.e.*, erythrocytes, leukocytes, platelet), circulating leukocyte subpopulations, hepatic enzymes (*i.e.*, transaminases, gamma-glutamyl transferase), and creatinine.

No signs of surgical site infection were observed during the first 2 months after surgery. In 1/17 (6.3%) rabbit, the surgical site was swollen and reddened during the 3^rd^ month after surgery. Since this alteration did not resolve with antibiotic therapy, the site was punctured, and 1 mL of purulent material was drained. After drainage, the surgical site recovered uneventfully.

## Discussion

### Bioengineered scaffolds outperformed the spontaneous bone regeneration process

The present preclinical study demonstrated that bone regeneration in the mandible is faster and more efficient when a scaffold composed of either HyCh or PLA-PCL-HyCh seeded with hMSCs is placed in the bony defect. In particular, HyCh with hMSCs was associated with the best performance, with density of the surgical site, measured with *in vivo* imaging, reaching roughly 50%-to-70% of the native density at 2–4 months after surgery. PLA-PCL-HyCh with hMSCs also showed excellent performance, with roughly 40%–60% of the native density being restored over the same time span. Of note, both these bioengineered materials outperformed controls with no reconstruction, where spontaneous bone regeneration took place. Interestingly, when focusing on unseeded scaffolds, only PLA-PCL-HyCh was associated with an improvement in terms of RDI, whereas HyCh showed a bone regeneration performance that was similar to non-reconstructed controls. This might be related to the intrinsic osteogenic properties of PLA-PCL in contrast to HyCh (Hwang et al., 2013; Narayanan et al., 2016; Teoh et al., 2019). However, HyCh was associated with the highest enhancement of the surgical site in the long term, which is consistent with the belief that HyCh promotes neovascularization. The molecular profile of newly formed bone also corroborated an active role played by scaffolds in the regeneration process. Osteopontin expression was higher in defects implanted with a scaffold. This sialoprotein not only is expressed in differentiated cells of the osteogenic lineage such as osteoblasts and osteocytes, but also is a marker for bone remodeling which is essential to new bone formation and maintenance of adequate bone quality (Depalle et al., 2021). VEGF-A was more expressed in PLA-PCL-HyCh-reconstructed defects than HyCh-reconstructed and not reconstructed ones. VEGF-A is expressed and secreted in response to poor tissue oxygenation, which depends upon vascularity (Hu and Olsen, 2016). The fact that HyCh-reconstructed sites were associated with the lowest expression of VEGF-A could mean that tissues within those surgical sites were adequately oxygenated and is consistent with the pro-angiogenetic properties of this material.

These results reinforced the belief that different properties of HyCh and PLA-PCL-HyCh should be exploited to optimize the functionality of a bioengineered, bone-regenerative medical device. Besides merging PLA-PCL and HyCh at a microstructural level, creation of composite scaffolds with hybrid macrostructure including a PLA-PCL-HyCh framework with interspersed pure HyCh areas is a step forward in bone regeneration.

There is significant proof that hMSCs play an essential role in the bone regeneration process observed, which is consistent with other observations (Cecilia and Maria, 2014; Pilipchuk et al., 2015; Saeed et al., 2015; Hosseinpour et al., 2017; Kim et al., 2017; Roffi et al., 2017; Ho-Shui-Ling et al., 2018). The presence of hMSCs significantly increased relative density restoration, with seeding concentration of 3,000 cells/mm^3^ being associated with the best performance. Although no effect on enhancement could be demonstrated when considering seeded scaffolds altogether, a clear increase in surgical site enhancement was associated with the 3,000 cells/mm^3^ group. These findings are consistent with the well-known osteogenic potential and pro-angiogenetic effect of hMSCs and suggest that 3,000 cells/mm^3^ is the optimal concentration among those studied herein (Yang et al., 2014; Ulpiano et al., 2021). Interestingly, cells staining positive for the human nuclear antigen were observed in the surgical site several months after surgery and were found to be more frequent in the 2,000 and 3,000 cells/mm^3^ group compared with controls and the 1,000 cells/mm^3^ group. No information on cell differentiation was gathered. Therefore, this observation mandates further investigation, but might confirm that hMSCs do not act as simple bystanders or initial triggers, but could have integrated in the host and possibly coordinated the regeneration process for a relatively long period.

Finally, it is worth specifying that timing and entity of density restoration is probably inappropriate for the purpose of translating these scaffolds to the clinical setting. Optimization of the regenerative performance is indeed paramount, and the results presented here will establish a baseline reference for future experiments from our collaborative research group. Other groups have adopted promising strategies including use of ossification-triggering factors (*e.g.*, bone morphogenic proteins, HMGB-1) (Cecilia and Maria, 2014; Pilipchuk et al., 2015; Saeed et al., 2015; Hosseinpour et al., 2017; Kim et al., 2017; Roffi et al., 2017; Ho-Shui-Ling et al., 2018; Monir et al., 2021) co-culture of endothelial progenitors, use of pedicle including scaffold (Shanbhag et al., 2017; Chen et al., 2019).

### Analysis of translationally relevant variables showed favorable results

The first translationally relevant variable analyzed in the present study was defect size. Mandibular defects requiring reconstruction in humans are usually large and include several mandibular segments among symphysis, parasymphysis, body, and ramus. While there is no universally accepted cutoff to define critical size defects in the rabbit’s mandible, the defects created in the present study can be considered non-critical in size, which means that this experimental defect is supposed to spontaneously heal over a given time (Young et al., 2008; Shah et al., 2016). The standard defect in the inferior aspect of the mandible was bi-cortical, three-dimensional, and had a volume of 45 mm^3^ and drilled bony surface of 33 mm^2^ in the defect bed. Other authors described a critical size defect created through a bi-cortical circular trephine with 1 cm diameter, which, considering a mean mandibular body thickness of around 5–7 mm, has a volume of 393–550 mm^3^ with a drilled bony surface of 157–220 mm^2^ in the defect bed (Young et al., 2008; Shah et al., 2016). Defects labelled as “large” in the present study were 3 times as large as small ones (135 mm^3^ *vs* 45 mm^3^; drilled bony surface in the defect bed 63 mm^2^ *vs* 33 mm^2^), but still did not reach the critical size volume. However, since bone regeneration is hypothesized to start from the bony edges of the defect, the surface of healing bone (*i.e.*, the drilled bony surface in the surgical bed) should also be considered to genuinely define critical size defects. Periosteal removal and cauterization of defect edges should also be considered as factors challenging bone regeneration (Carlisle et al., 2019). Irrespective of the non-critical size of the defects studied herein, it should be noted that non-reconstructed defects did not heal completely over 4 months. Most importantly, the study groups of defects reconstructed with bioengineered scaffolds showed a faster and more efficient bone regeneration process. In addition to defect size, the segmental *vs* marginal nature of a mandibular defect is of utmost importance from a clinical perspective and can be studied in rabbits (Hirota et al., 2016). A segmental defect, indeed, implies that the mandible is discontinued, thus requiring that the reconstruction can substitute for the mechanical function of the bone over the healing period. This aspect was not investigated in the present study and will represent the object of future research from our groups. Of relevance, large defects did not show a significantly reduced RDI as compared to small defects, which means that, within the dimensional range of defects studied here, defect size did not impact the performance of bone regeneration at a densitometric level. Deeper and larger defects will be assessed in our future research to address this issue. Of note, large defects showed reduced enhancement in the surgical site, particularly in the early postoperative period. Re-vascularization of the surgical site might indeed be slowed in large surgical sites. Given the crucial role of adequate vascularization to sustain bone regeneration, this issue should be considered for clinical translation, particularly if adopting intramembranous ossification-based strategies (like the one employed herein), which are associated with less robust re-vascularization as opposed to endochondral ossification-based ones (Lopes et al., 2018). TRAP was found to be less expressed in large defects. Besides marking osteoclasts, TRAP is expressed by other cells in bone regeneration (*e.g.*, TRAP-positive mononuclear cells), whose presence is considered a hallmark of active bone regeneration via periosteum-derived cells recruitment (Gao et al., 2019). Thus, slightly less efficient bone regeneration in large defects was unveiled through immunohistochemistry and contrast-enhanced imaging. These findings further underline that regenerative strategies oriented towards large bony defects should be sensitive to re-vascularization of the surgical site. This is emphasized by the observation by Chen *et al.* that co-culturing endothelial progenitors with mesenchymal stromal cells, channeling the scaffold to promote neo-angiogenesis, and incorporating vessels in the scaffold were all effective strategies in long bone reconstruction (Chen et al., 2019; Vidal et al., 2020).

The second translationally relevant variable analyzed in the present study was the defect site. Mandibular defects are most often created through clean-contaminated fields, which means that the reconstruction is temporarily in contact with saliva and oral microbes and is thus partially contaminated. Also, orocutaneous fistula can occur during the postoperative period, thus leading saliva and microbes to the healing surgical site. In contrast to reconstruction with free tissue transfer, which, being vascularized, benefit from immune system defense, scaffolds are prone to potential microbic contamination, which represents a relevant concern and is partially responsible for preventing their translation into clinical practice. In the present study, defects created through a clean-contaminated field did not show a reduced performance of bone regeneration, nor did they show signs of infection in the postoperative period. Defects on the oral aspect of the mandible showed higher RDI, enhancement, and bone sialoprotein expression and lower osteopontin and osteocalcin expression compared with other experimental subgroups. Bone sialoprotein is thought to play a role in bone regeneration-related neovascularization (Bellahcène et al., 2000), which is consistent with the finding of increased enhancement in defects located in the oral aspect of the mandible. These findings are cautiously encouraging in a perspective of performing scaffold-based reconstruction of the mandible. Nonetheless, besides contamination occurring during surgery, the mucosal wound was closed at the end of the procedure, which means that the surgical site was no longer in contact with potential sources of contamination in the postoperative period. This issue will be assessed in future experiments in order to analyze the consequences of prolonged contact of the scaffold with saliva and oral microbes.

The third translationally relevant variable analyzed in the present study was shape restoration, which is of primary importance in the field of craniofacial reconstruction. This issue was assessed through part-comparison analysis, a method quantifying the morphological similarity between 2 objects, which is best expressed by means of RMS (*i.e.*, the lower is RMS the higher is morphological similarity) (Pagedar et al., 2012b). By comparing the cortical surface of the healing surgical site with the native cortical bone throughout the course of the study, the timing and the contour of the shape restoration could be measured. Cortical shape restoration was significantly more pronounced in defects located on the oral aspect of the mandible, which were though the smallest of the series, and in cases with 2,000–3,000 cells/mm^3^ at the time of seeding (13%–19% RMS increase with respect to control groups). While firm conclusions cannot be drawn based on these preliminary results, scaffolds and hMSCs might have played a role in favoring shape restoration. This issue should be investigated in larger and morphologically more complex defects.

### Microarchitectural bone features were not completely restored by any regeneration process

The microarchitectural features assessed in the present study included relative bone volume, trabecular thickness, trabecular density, and trabecular separation, which are all essentially associated with mechanical properties of the bone (Mittra et al., 2005). While trabecular density and separation were not significantly different when comparing the study subgroups with a group of non-operated rabbits, relative bone volume and trabecular thickness were significantly reduced in regenerated bone irrespective of the reconstructive strategy. Interestingly, the only measurements equating the native bone microarchitecture in terms of relative bone volume and trabecular thickness were in the group of rabbits receiving seeded scaffold-based reconstruction (Figure 11). These findings suggest that the majority of regenerated bone areas were biomechanically inferior to the native bone around 4 months after surgery. Although this does not necessarily mean that regenerated bone is biomechanically inadequate to sustain mandibular functions such as chewing, it is logical to assert that microarchitectural bone features should be an additional outcome to be considered in future optimization of our and other bone regenerative devices. Potential improvement of microarchitectural evaluation in future research could include the measurement of bone stiffness and ultimate load, a method used for virtual biomechanical analyses of peripheral bone sites such as the distal segment of radius and tibia (Schenk et al., 2022).

### Safety assessment

Overall, the experimental procedure presented here, including the surgery, synthetic material implantation, and xenograft, were relatively safe. Mortality was 5.9% within 1 month from surgery, which compares favorably with other results (33.3%) reported for segmental defects in rabbit mandibles (Lopez et al., 2018). The only case of early death was observed in a rabbit secondary to pneumonia with atelectasis. No bronchial foreign body was found at autopsy, nor did the latest white blood cell count suggest systemic immune deficiency. Another rabbit was found dead in the cage 71 days after surgery. Autopsy showed pulmonary hemorrhage, cardiomegaly, and coronary thrombosis, which suggested an acute myocardial ischemia with heart failure. A clear relationship with the experimental procedure could not be established in either of these cases. However, it is worth mentioning that coagulation time was not tested in this study.

In terms of infection of the surgical site, only a late event was observed. A small abscess was found 3 months after surgery in a rabbit that underwent a large mandibulectomy. Despite the time passed from surgery, a potential role played by the scaffold in determining or facilitating the surgical site infection could not be excluded.

### Limitations and strength of the study

The present study has limitations worth being commented on. First, the sample size is limited and numerosity of subgroups is low. This owes to the pilot nature of the study but confers some degree of uncertainty to several results reported herein. All findings reported in this study should thus be intended as preliminary and will be validated in future analyses. Second, a xenograft model including scaffolds seeded with human cells implanted into rabbits was adopted. Poor immunogenicity of hMSC justifies this approach, and lack of rejection-related adverse events proved its safety. However, this strategy implies differences to what would theoretically be done in humans, posing limitations in terms of clinical translation. Third, although *in vivo* imaging is regularly used in preclinical studies on animals, it comes with limitations related to the biological interpretation of findings. For instance, the main outcome of the present study was RDI. However, a variety of mechanisms determining RDI can be hypothesized from a biological standpoint, including ossification and calcification. On the other hand, this study has the strengths of using cutting-edge materials and being one of the first preclinical analyses assessing clinically relevant variables that are critical to head and neck surgery.

The regenerative model presented herein does not meet the clinical needs of craniofacial defects reconstruction yet. However, one can hypothesize that if performance of the model is enhanced in terms of quantity, quality, and timing of new bone formation, then a fully or hybrid (*i.e.,* combination with free tissue transfer) bioengineered reconstruction of craniofacial defects might take place (Ismail et al., 2021). Indeed, a preliminary preclinical report on feasibility of bioengineered reconstruction of craniofacial defects using computer-aided design 3D-printed polymeric scaffolds in cadaver models has been recently published (Mattavelli et al., 2024).

## Conclusion

The present preclinical study demonstrated that bone regeneration in the rabbit mandible can be boosted by scaffold composed of either HyCh or PLA-PCL-HyCh seeded with hMSCs. Compared to spontaneous regeneration of bone, which led to approximately 40% restoration of the presurgical bone density in around 120 days, scaffold- and seeded scaffold-reconstruction increased this outcome to roughly 50% and 70%, respectively. HyCh was associated with increased enhancement of the surgical site over time, and PLA-PCL-HyCh with spontaneous osteogenic activity from the unseeded scaffold. Several results suggest a significant role of hMSCs, whose presence in the scaffold was associated with increased relative density, enhancement, and shape restoration, particularly at a concentration at the time of scaffold seeding of 2,000–3,000 cells/mm^3^. Native microarchitectural characteristics were not demonstrated in any experimental group. Overall, the experimental procedure was safe and not associated with adverse events relatable to scaffolds or xenotransplantation. Next step in research will address the optimization of the model, seeking for an efficient synergy between the three key elements of bone regeneration (*i.e.,* scaffold, cells, and growth stimuli). For instance, data on growth stimuli, such as BMP-2 and prostaglandin derivatives, and co-culturing approaches are particularly promising (Chen et al., 2019; Sheikh et al., 2020; Kudaibergen et al., 2024).

## Data Availability

The raw data supporting the conclusions of this article will be made available by the authors, without undue reservation.
