# Supplementary material for: Hydrogel-chitosan and polylactic acid-polycaprolactone bioengineered scaffolds for reconstruction of mandibular defects: a preclinical in vivo study with assessment of translationally relevant aspects
Source: Front Bioeng Biotechnol. 2024 Jul 15;12:1353523. doi: 10.3389/fbioe.2024.1353523 (PMC11284118; doi:10.3389/fbioe.2024.1353523)
Supplement: Supplementary file 1 [file Table1.DOCX]

**Supplementary Tables**

| **Variable(s)** | **Site of the defect** | **Size of the defect** | **Material** | **Material and seeding status** | **Material and cell concentration** |
| --- | --- | --- | --- | --- | --- |
| **Clustering** | Cervical: 20  Oral: 4 | Small: 20   - 45 mm^3^: 16 - 27 mm^3^: 4   Large (135 mm^3^): 4 | HyCh: 10  PLA-PCL-HyCh: 11  None: 3 | HyCh: 1  HyCh + hMSCs: 9  PLA-PCL-HyCh: 2  PLA-PCL-HyCh + hMSCs: 9  None: 3 | HyCh: 1  HyCh + 1K-hMSCs: 1  HyCh + 2K-hMSCs: 4  HyCh + 3K-hMSCs: 4  PLA-PCL-HyCh: 2  PLA-PCL-HyCh + 1K-hMSCs: 1  PLA-PCL-HyCh + 2K-hMSCs: 4  PLA-PCL-HyCh + 3K-hMSCs: 4  None: 3 |

**Table S1**. Subgroup clustering according to main explanatory variables and their combination. Numbers refer to the surgical defects. hMSC, human mesenchymal stromal cell; HyCh, hydrogel-chitosan scaffolds; PLA-PCL-HyCh, polylactic acid-polycaprolactone-hydrogel chitosan scaffolds.

| **Clustering variable** | **60-day enhancement (HU)** | **120-day enhancement (HU)** | **p-value*** |
| --- | --- | --- | --- |
| None (entire series) | 151.5 | 176.2 | N.A. |
| Scaffold (no *vs* yes) | No: 219.0  Yes: 143.4 | No: 107.5  Yes: 178.9 | 0.0817 |
| Scaffold type (no recon. *vs* HyCh *vs* PLA-PCL-HyCh) | No recon.: 224.1  HyCh: 185.8  PLA-PCL-HyCh: 102.5 | No recon.: 144.4  HyCh: 216.0  PLA-PCL-HyCh: 139.5 | **0.0007** |
| Scaffold seeding status (no recon. *vs* seeded scaffold *vs* unseeded scaffold) | No recon.: 224.1  Seeded scaffold: 136.4  Unseeded scaffold: 189.2 | No recon.: 144.4  Seeded scaffold: 170.1  Unseeded scaffold: 247.0 | 0.0779 |
| Scaffold type and seeding status (no recon. *vs* HyCh ± hMSCs *vs* PLA-PCL-HyCh ± hMSCs) | No recon.: 224.1  HyCh alone: 303.3  HyCh + hMSCs: 174.4  PLA-PCL-HyCh alone: 126.5  PLA-PCL-HyCh + hMSCs: 98.6 | No recon.: 224.1  HyCh alone: 311.5  HyCh + hMSCs: 206.9  PLA-PCL-HyCh: 200.8  PLA-PCL-HyCh + hMSCs: 132.8 | **0.0008** |
| Defect site (no seeding/no scaffold *vs* cervical *vs* oral) | No seeding/no scaffold: 206.7  Cervical: 115.7  Oral: 223.6 | No seeding/no scaffold: 195.7  Cervical: 151.8  Oral: 236.9 | **0.0006** |
| Defect size (no seeding/no scaffold *vs* small *vs* large) | No seeding/no scaffold: 206.7  Small: 147.9  Large: 91.8 | No seeding/no scaffold: 195.7  Small: 177.2  Large: 149.2 | **0.0274** |
| hMSCs concentration (no cells *vs* 1000 cells/mm^3^ *vs* 2000 cells/mm^3^ *vs* 3000 cells/mm^3^) | No seeding/no scaffold: 206.7  1000 cells/mm^3^: 108.8  2000 cells/mm^3^: 108.5  3000 cells/mm^3^: 172.7 | No seeding/no scaffold: 195.7  1000 cells/mm^3^: 147.2  2000 cells/mm^3^: 144.4  3000 cells/mm^3^: 204.2 | **0.0136** |

**Table S2**. Estimates of enhancement of the surgical site, measured in Hounsfield units (HU), at 60 and 120 days after surgery, clustered by explanatory variables considered in the study. *The p-value refers to the analysis of variance test (ANOVA) on linear regression models, see the text for relevant *post hoc* pairwise comparisons between categories. hMSC, human mesenchymal stromal cell; HyCh, hydrogel-chitosan scaffolds; PLA-PCL-HyCh, polylactic acid-polycaprolactone-hydrogel chitosan scaffolds.

| **Clustering variable** | **60-day RMS (mm)** | **120-day RMS (mm)** | **p-value*** |
| --- | --- | --- | --- |
| None (entire series) | 0.77 | 0.65 | N.A. |
| Scaffold (no *vs* yes) | No: 0.78  Yes: 0.77 | No: 0.69  Yes: 0.64 | 0.8087 |
| Scaffold type (no recon. *vs* HyCh *vs* PLA-PCL-HyCh) | No recon.: 0.78  HyCh: 0.76  PLA-PCL-HyCh: 0.78 | No recon.: 0.69  HyCh: 0.64  PLA-PCL-HyCh: 0.64 | 0.9239 |
| Scaffold seeding status (no recon. *vs* seeded scaffold *vs* unseeded scaffold) | No recon.: 0.78  Seeded scaffold: 0.76  Unseeded scaffold: 0.81 | No recon.: 0.69  Seeded scaffold: 0.63  Unseeded scaffold: 0.74 | 0.5312 |
| Scaffold type and seeding status (no recon. *vs* HyCh ± hMSCs *vs* PLA-PCL-HyCh ± hMSCs) | No recon.: 0.78  HyCh alone: 0.92  HyCh + hMSCs: 0.75  PLA-PCL-HyCh alone: 0.76  PLA-PCL-HyCh + hMSCs: 0.78 | No recon.: 0.69  HyCh alone: 0.69  HyCh + hMSCs: 0.63  PLA-PCL-HyCh: 0.79  PLA-PCL-HyCh + hMSCs: 0.63 | 0.4747 |
| Defect site (no seeding/no scaffold *vs* cervical *vs* oral) | No seeding/no scaffold: 0.79  Cervical: 0.80  Oral: 0.61 | No seeding/no scaffold: 0.72  Cervical: 0.76  Oral: 0.54 | **0.0006** |
| Defect size (no seeding/no scaffold *vs* small *vs* large) | No seeding/no scaffold: 0.79  Small: 0.76  Large: 0.79 | No seeding/no scaffold: 0.72  Small: 0.63  Large: 0.63 | 0.5258 |
| hMSCs concentration (no cells *vs* 1000 cells/mm^3^ *vs* 2000 cells/mm^3^ *vs* 3000 cells/mm^3^) | No seeding/no scaffold: 0.79  1000 cells/mm^3^: 0.93  2000 cells/mm^3^: 0.73  3000 cells/mm^3^: 0.75 | No seeding/no scaffold: 0.72  1000 cells/mm^3^: 0.80  2000 cells/mm^3^: 0.58  3000 cells/mm^3^: 0.63 | **0.0007** |

**Table S3**. Estimates of root mean square (RMS) of the part-comparison-analysis with respect to the preoperative cortical anatomy at 60 and 120 days after surgery, clustered by explanatory variables considered in the study. *The p-value refers to the analysis of variance test (ANOVA) on linear regression models, see the text for relevant *post hoc* pairwise comparisons between categories. hMSC, human mesenchymal stromal cell; HyCh, hydrogel-chitosan scaffolds; PLA-PCL-HyCh, polylactic acid-polycaprolactone-hydrogel chitosan scaffolds.
